# Biocompatible Preparation of Beta-Lactoglobulin/Chondroitin Sulfate Carrier Nanoparticles and Modification of Their Colloidal and Hydropathic Properties by Tween 80

**DOI:** 10.3390/polym16141995

**Published:** 2024-07-12

**Authors:** Ioannis Pispas, Nikolaos Spiliopoulos, Aristeidis Papagiannopoulos

**Affiliations:** 1 Theoretical and Physical Chemistry Institute, National Hellenic Research Foundation, 48 Vassileos Constantinou Avenue, 11635 Athens, Greece; johnpispas@gmail.com; 2 Department of Physics, University of Patras, 26504 Patras, Greece; nspiliop@physics.upatras.gr

**Keywords:** nanoparticles, bovine beta-lactoglobulin, porcine chondroitin sulfate, electrostatic complexation, thermal treatment, Tween 80

## Abstract

The electrostatic complexation of the protein beta-lactoglobulin (β-LG) with the anionic polysaccharide chondroitin sulfate (CS) and the subsequent stabilization by thermal treatment were studied to achieve the well-defined nanoparticles (NPs). The formation of the well-defined NPs was obtained at pH 4 with a hydrodynamic radius from 60 to 80 nm. NP aggregation was observed at pH 1.5 because of the loss of the anionic charge of chondroitin sulfate on the surface of the NPs. After thermal treatment, the NPs exhibited stability against a pH increase to pH 7 while a stronger aggregation at pH 1.5 was observed. Core-shell structures were found at pH 7 after thermal treatment, indicating a possible mechanism of partial disintegration. The addition of Tween 80 (T80) before thermal treatment led to the formation of T80 self-assemblies inside the NPs. This caused an increase in the hydrophobicity of the inner and outer surfaces of the NPs as it was observed by fluorescence spectroscopy. The ζ-potential of the complexes and NPs was about −20 mV while the presence of T80 did not affect it. FTIR spectra verified changes of the secondary structure of β-LG in its complexes with CS and T80. The thermally treated NPs exhibited high surface and overall hydrophobicity and stability in high salinity and biocompatible solutions. The thermally treated NPs showed colloidal and physicochemical stability for 1 month, which were enhanced by the addition of T80. Due to the nature of the precursors and their colloidal properties, the NPs are highly promising for applications as biocompatible drug delivery nanocarriers while T80 acts as an agent to modify their properties.

## 1. Introduction

Recent advances in nanotechnology have led to the development of a large variety of new materials in medical science, food science and pharmaceutics. Especially for drug delivery and drug targeting, polymeric nanoparticles (NPs) are highly suitable [1,2]. Polymeric NPs are NPs derived from biocompatible and biodegradable polymers, such as biological macromolecules, with sizes ranging from 1 to 1000 nm [3]. Polymeric NPs are generally stimuli responsive and may interact with both hydrophobic and charged substances. A variety of techniques exists for preparing PNPs, with two of them involving the association of amphiphilic block polyelectrolytes by self-assembly into micellar nanostructures [4,5] and of oppositely charged polyelectrolytes by complexation and coacervation [6]. Examples of the latter case are polyelectrolyte/protein complexes and more specifically polysaccharide/protein complexes, which rely on the properties of both polysaccharides and proteins, the pH-dependent surface charge distribution of the proteins and are sensitive to external stimuli such as ionic strength, temperature and others [7,8,9]. The choice of proteins in these systems relies on their ability to act as versatile nanocarriers and their nature as complex macromolecules with varying surface charges and hydrophobic domains in relation to external parameters [10,11,12,13,14]. On the other hand, polysaccharides are biocompatible and biodegradable with high stability and no toxicity, rendering them a suitable option for complexation with proteins such as globular proteins [15]. In addition, surfactants can be used because they can adsorb at amphiphilic interfaces to form hydrophobic regions inside the nanostructures and strengthen the encapsulation of hydrophobic compounds [16,17].

Beta-lactoglobulin (β-LG) is a whey protein originating primarily from mammal milk such as bovine or cow milk [18,19]. It is capable of binding various hydrophobic molecules and ligands including antioxidants and vitamins and significantly increases their overall transport in biological systems [18,20]. It belongs to the larger family of lipocalin proteins and, as a whey protein, it is globular. An exception to this case is human breast milk, which lacks a certain homologue [21]. Even though the homologue of β-LG is absent in human breast milk, the protein has the ability to transfer iron molecules into human cells, providing micronutrition to the cells and enhancing the immune system [19]. β-LG-based NPs have been produced as carriers of caffeine [22] and as stabilizers and enhancers in insulin nanodelivery [23]. Chondroitin sulfate (CS) is an anionic polysaccharide that belongs to the family of sulfated glycosaminoglycans (GAGs) [24]. Its basic disaccharide unit consists of N-acetylgalactosamine and glucuronic acid [25]. CS is considered an important dietary supplement for therapy of many patients suffering from osteoarthritis [26], alongside other compounds, such as glucosamine [27]. CS is usually acquired from bovine, porcine or marine cartilage, where it acts as a structural component [28,29] forming the side chains of aggrecan [30]. Tween 80 (T80) is a well-known non-ionic surfactant and emulsifier that has been successfully used in vitamin D delivery by mixed surfactant food-grade emulsions [31], in anticancer drug delivery [32] and in skincare formulations [33]. It is a relatively cheap compound with a distinct yellow discoloration and low toxicity levels, due to its non-ionic nature. Some of its most noted remarks are its ability of protein stabilization against thermal denaturation, aggregation and even surface adsorption [34].

β-LG in combination with low-methoxyl pectin (LMP) has been used in electrostatic protein/polysaccharide complexes to produce well-defined NPs (50–70 nm in size) and encapsulate the model hydrophobic nutraceutical vitamin D_2_ (VD_2_) [35]. The protein is responsible for transporting and securing VD_2_ to some degree while β-LG/LMP NPs were able to provide protection of VD_2_ against degradation. Few records of protein/polysaccharide/surfactant systems exist, where in one case, zein/propylene glycol alginate (PGA) with soy lecithin were fabricated for the encapsulation of curcumin [36]. The presence of the surfactant increased the encapsulation efficiency, as well as the protection and delivery of curcumin in contrast to the usage of binary protein/polysaccharide complexes. In another study, both the catalytic activity and thermal stability of the engineered allosteric enzyme MPB317-347 were enhanced by alginate/lysozyme NPs when lecithin was present [37].

In the present study, the electrostatic complexation of β-LG and CS in acidic conditions was investigated to fabricate well-defined biocompatible NPs. The size, mass and ζ-potential were measured using light scattering methods and transmission electron microscopy, and the hydrophobicity was investigated using fluorescence spectroscopy. Thermally treating β-LG inside the electrostatic complexes resulted in the thermal stabilization of the NPs (i.e., their resistance to increases of pH). The addition of T80 was studied using light scattering methods and fluorescence spectroscopy. It was found that the binding of the surfactant influenced both the stability and the hydrophobicity of the NPs. Circular dichroism and FTIR were used to determine changes in the secondary structure of β-LG. The NPs were tested in solutions of added salt and biological fluids, thereby showing a high possibility of being utilized as biocompatible delivery nanosystems.

## 2. Materials and Methods

### 2.1. Materials and Sample Preparation

Porcine chondroitin sulfate (CS) in the form of sodium salt (Na-CS) was purchased from Bioiberica (Barcelona, Spain), while bovine beta-lactoglobulin (β-LG) and Tween 80 (T80) were purchased from Sigma-Aldrich (Burlington, MA, USA) and were used without additional treatment. NaCl, NaOH, HCl, citric acid (CA), pyrene, 8-Anilino-1-naphthalenesulfonate (ANS), phosphate buffer saline (PBS) and fetal bovine serum (FBS) were purchased from Sigma-Aldrich (Burlington, MA, USA). Solutions of CA in distilled water were prepared at pH 4 and used as a solvent for all the stock solutions. The stock solutions were prepared under stirring at concentrations of 1.0 mg/mL each at pH 4 and kept for 20–24 hours at approximately 4 °C to reach equilibrium. The β-LG/CS/T80 complexes were prepared after mixing volume ratios of the stock solutions of β-LG, CS, T80 and solvent, while maintaining the final β-LG concentration at 0.1 mg/mL (unless stated differently). The order of addition involved the addition of water, followed by CS, then β-LG and, lastly, T80 when present in the solutions. The total volume of the solutions was 1 mL in all cases. Small amounts of aqueous solutions of NaOH and HCl (1 M each) were used to adjust the pH of the complexes’ solution from pH 4 to neutral (pH 7) or to strongly acidic (pH 1.5). Thermal treatment (TT) of the β-LG and complexes was performed following a heating protocol of 85 °C for 50 minutes. The samples were thermally treated at pH 4 after the formation of the complexes. After TT, the samples were slightly stirred and left to cool at 25 °C.

NaCl was used to regulate the salt concentration. Solutions of FBS, prepared in PBS (FBS:PBS, volume ratio of 1:9) after the use of 0.45 μm pore size hydrophilic sartorius filters, were employed for studies in biological fluids. The NPs solutions were mixed with 10 μL of ANS dispersion (5 mM) in distilled water for surface hydrophobicity experiments and were placed in the refrigerator for 1 day at 4 °C to equilibrate. In the hydrophobicity experiments with pyrene, 2 μL pyrene were added to the NPs solutions and the samples were stored for 1 day at 4 °C to attain equilibrium.

For circular dichroism (CD) experiments, NPs were prepared with a β-LG concentration of 0.2 mg/mL to obtain a satisfactory signal. For transmission electron microscopy (TEM) experiments, the samples were prepared with a β-LG concentration of 0.2 mg/mL. All the experiments were conducted at 25 °C. The presented results are in the form of mean values and standard deviation after executing them three times minimum.

### 2.2. Surface Charge, Hydropathy and Secondary Structure Calculations Based on the Crystal Structure of β-LG

The crystal structure of bovine β-LG was provided by the Research Collaboratory for the Structural Bioinformatics Protein Data Base (RCSB PDB). The PDB ID 3NPO for the atomic coordinates of the folded protein was used and the PROKPA method was applied in conjunction with the PDB2PQR software to determine the dissociation constants ${\mathrm{pK}}_{a}^{i}$ of each chargeable amino acid and the charge of each amino acid utilizing the Henderson–Hasselbalch equation [38,39].

The Adaptive Poisson–Boltzmann Solver (APBS) software was employed for the evaluation of the surface electrostatic potential of β-LG by solving the Poisson–Boltzmann equation. It is an open source and free software designed to solve the equations of the continuum electrostatics for large biological macromolecules [40].

The hydropathy of the protein was computed with the protein-sol patches web software. Patch analysis is used in the analysis of nonpolar surfaces, and benchmark analysis of β-LG is crucial in demonstrating and studying the environments of the tryptophan residues of the protein. The algorithm compares the solvent accessible surface area (SASA) ratio of nonpolar to polar (NPP ratio) for all nonhydrogen atoms to determine the degree of surface patch hydrophobicity of the protein. Knowledge related to the hydropathy of the environments and regions around tryptophan residues can be useful in the assessment of the complexation with the anionic CS and the binding of T80 [41].

The Secondary Structure Server (2Struc) was used to extract the protein’s secondary structure from its documented crystal structure PDB ID 3NPO where the amino acid coordinates of β-LG were measured using X-ray crystallography at 2.20 Å. The simplified three-state representation was chosen, given as helix (including H: α-helix, I: π-helix, and G: 3–10 helix), sheet (E: β-strand) and others (T: turns, S: bends, B: β-bridge, and -: irregular/loop). The 2Struc calculates the percentage of eight individual secondary structure elements (SSE). H, I, G and E are taken from their respective Define Secondary Structure of Proteins (DSSP) definitions. The three-state SSE assignments for each method are compared to DSSP using multiple commonly used metrics, including percentage similarity, the Matthews correlation coefficient (MCC) and the MCC squared [42,43].

### 2.3. Static and Dynamic Light Scattering

For static and dynamic light scattering (SLS and DLS) measurements, a classic ALV system (ALV GmbH, Hessen, Germany) was employed, executing an analysis via an ALV-CG-3 goniometer and an ALV-5000/EPP multi-tau digital correlator with a He-Ne laser (λ = 632.8 nm). In SLS, the time-averaged scattered intensity $\langle I\left(q\right)\rangle$ is reduced to the Rayleigh ratio $R\left(q\right)$ [44,45]. The average apparent molecular mass M and the form factor $P\left(q\right)$ are connected by Equation (1)

(1) $$\frac{\mathrm{Kc}}{R\left(q\right)}=\frac{1}{\mathrm{MP}\left(q\right)}$$

where c is the mass solution concentration, q is the scattering wave vector ($q=\frac{4{\pi n}_{o}}{\lambda }\sin\left(\frac{\theta }{2}\right)$) and K is the light scattering (LS) contrast factor ($K=\frac{4\pi^{2}n_{o}^{2}}{N_{A}\lambda^{4}}{\left(\frac{\partial n}{\partial c}\right)}^{2}$). In the expression for q, λ is the wavelength of the laser, $n_{o}$ is the refractive index of the solvent and θ is the scattering angle. In the expression for K, $N_{A}$ is the Avogadro number and $\frac{\partial n}{\partial c}$ is the refractive index increment of the solute and solvent system. In the present study, the values of ${\left(\frac{\partial n}{\partial c}\right)}_{\beta -\mathrm{LG}}=0.185\;\mathrm{mL}/g$ [46] and ${\left(\frac{\partial n}{\partial c}\right)}_{\mathrm{CS}}=0.143\;\mathrm{mL}/g$ [47] were used, while for T80, its refractive index increment ${\left(\frac{\partial n}{\partial c}\right)}_{T80}\approx \frac{n_{T80}-n_{o}}{d_{T80}}$ ($n_{T80}=1.47,{\;d}_{T80}=1.064\;g/\mathrm{mL}$) is estimated at 0.132 mL/g. Using Equation (2), the values of $\frac{\partial n}{\partial c}=0.173\;\mathrm{mL}/g$ and $\frac{\partial n}{\partial c}=0.162\;\mathrm{mL}/g$ were calculated for the cases of samples without (excluded the third term in Equation (2)) and with T80, respectively. Therefore, an estimated value of $\frac{\partial n}{\partial c}=0.17\;\mathrm{mL}/g$ is used for all cases of mass ratios in the present research work

(2) $$\frac{\partial n}{\partial c}=\frac{c_{\beta -\mathrm{LG}}}{c_{\beta -\mathrm{LG}}+c_{\mathrm{CS}}+c_{T80}}{\left(\frac{\partial n}{\partial c}\right)}_{\beta -\mathrm{LG}}+\frac{c_{\mathrm{CS}}}{c_{\beta -\mathrm{LG}}+c_{\mathrm{CS}}+c_{T80}}{\left(\frac{\partial n}{\partial c}\right)}_{\mathrm{CS}}+\frac{c_{T80}}{c_{\beta -\mathrm{LG}}+c_{\mathrm{CS}}+c_{T80}}{\left(\frac{\partial n}{\partial c}\right)}_{T80}$$

The Guinier approximation ($P\left(q\right)=e^{-\frac{1}{3}q^{2}R_{g}^{2}}$) was used for the form factor in Equation (1) where $R_{g}$ is the radius of gyration. To fit the whole series of the Guinier plots, a quadratic approximation with respect to $q^{2}$ is employed, as shown in Equation (3), due to polydispersity phenomenon in the solution (see Section 3) [48,49,50]

(3) $$\frac{R\left(q\right)}{\mathrm{Kc}}=M\times e^{-\frac{1}{3}q^{2}R_{g}^{2}+B{\left(q^{2}\right)}^{2}}$$

In DLS [51,52], the time-autocorrelation function of the scattered light intensity $g_{2}\left(q,\tau \right)$ is connected to the field autocorrelation function $g_{1}\left(q,\tau \right)$ through the Siegert relation (Equation (4))

(4) $$g_{2}\left(q,\tau \right)-1=\beta {\left|g_{1}\left(q,\tau \right)\right|}^{2}$$

where β is a normalization constant. The characteristic times of $g_{1}$ are related to the relaxation rates $\Gamma ={\mathrm{Dq}}^{2}\left(=1/\tau \right)$, where D is the diffusion coefficient and the hydrodynamic radius $R_{h}$ using the Stokes–Einstein equation (Equation (5))

(5) $$R_{h}=\frac{k_{B}T}{6\pi \eta D}$$

where $k_{B}$ is the Boltzmann constant, T is the temperature of the sample and η is the viscosity of the solvent. The shape factor $\rho =\frac{R_{g}}{R_{h}}$ provides significant information about the shape of the NPs. To analyze the distributions (scattered intensity-weighted) of the hydrodynamic radii at $\theta =90^{ο}$, the CONTIN analysis algorithm was utilized.

### 2.4. Electrophoretic Light Scattering

The electrophoretic light scattering (ELS) technique was performed using a Zetasizer Nano-ZS (Malvern Instruments Ltd., Malvern, UK). The device performs multiple measurements of the electrophoretic mobilities at a fixed angle of $\theta =173^{o}$, which is transformed into a zeta potential (ζ-potential) distribution. Ten measurements were taken at room temperature to determine the mean value and the standard deviation of the ζ-potential. It was calculated from the electrophoretic mobility $\mu_{e}$ (Equations (6) and (7)), under the Smoluchowski approximation, which implies that the value of Henry’s function $f\left(R/\lambda_{D}\right)$ is a constant equal to 3/2. In Henry’s function, R is the radius of the NPs and $\lambda_{D}$ is the Debye length

(6) $$\mu_{e}=\frac{v_{e}}{E}=\frac{2}{3}\frac{\varepsilon_{r}\varepsilon_{o}\zeta f\left(R/\lambda_{D}\right)}{\eta }$$

(7) $$\zeta =\frac{{\eta \mu }_{e}}{\varepsilon_{r}\varepsilon_{o}}$$

where $v_{e}$ is the drift velocity of the NP, E is the electric field, $\varepsilon_{r}$ and $\varepsilon_{o}$ are the relative dielectric permittivity of the solvent and the dielectric permittivity of the vacuum, respectively, and η is the viscosity of the solvent [53,54].

### 2.5. Fourier-Transform Infrared Spectroscopy

Fourier-transform infrared spectroscopy (FTIR) was performed using a device (Bruker Equinox 55, Ettlingen, Germany) with an attenuated total reflectance (ATR) diamond accessory (Dura Samp1IR from SENS-IR Technologies, Danbury, CT, USA). A few droplets (8 to 12 μL) of the solutions were placed onto the center of the holder of the samples and were dried under constant flow of nitrogen gas. Each spectrum was taken at least twice for reproducibility, completing a total of 64 scans with a resolution of 4 cm^−1^ in the range of 500 to 5000 cm^−1^.

For the evaluation of the secondary structure of the deconvolution of the amide I group from 1600 to 1700 cm^−1^, a code written in MATLAB for the nonlinear least-squares method was used, which determines the superposition of Gaussian peaks. The general formula, $f_{i}\left(x\right)=\frac{G_{i}}{\sigma_{i}\sqrt{2\pi }}e^{-\frac{{\left(x-x_{o,i}\right)}^{2}}{2\sigma_{i}^{2}}}$, consists of the absolute weight $G_{i}$, the standard deviation $\sigma_{i}$ and the position $x_{o,i}$ of each Gassian peak. Initial conditions for each of the three latter parameters were selected according to the experimental data of the FTIR spectra to assist the convergence of the algorithm. A single Gaussian peak or a pair of two is attributed to one secondary structure element. The percentage of each secondary structure element was computed by the relative weight of every Gaussian peak [50,55].

### 2.6. Fluorescence Spectroscopy

Fluorescence spectroscopy (FL) was performed using a Fluorolog-3 spectrofluorometer (model FL3-21, Jobin Yvon-Spex, Palaiseau, France) with a double-grating excitation, while a single-grating emission was engaged. A Xenon lamp was the light source and the excitation wavelengths λ = 370 nm for ANS, λ = 330 nm for pyrene and λ = 290 nm for the tryptophan (Trp) residues in the protein molecule were used. The range of spectra recording was 390–700 nm, 350–500 nm and 310–420 nm for ANS, pyrene and Trp, respectively. All measurements were set to an integration time of 0.5 s and the slit size was set to 2 nm.

### 2.7. Circular Dichroism

The circular dichroism (CD) technique was performed using a CD spectrophotometer (Jasco J-815 model, Tokyo, Japan) by placing the sample solutions into suprasil quartz cells of 1 mm thickness. The quartz cells were carefully cleaned using Milli-Q water, ethanol and nitrogen gas. CD spectra were recorded in the region of 185–250 nm and the data retrieved in units of mdeg were converted into molar ellipticity units Δε of M^−1^cm^−1^ (Equation (8))

(8) $$\Delta \varepsilon =\theta \cdot \frac{0.1\cdot \mathrm{MRW}}{\left(P\cdot c\right)\cdot 3298}$$

where θ is the measured ellipticity in mdeg, MRW is the mean residue weight, Δε is the molar ellipticity per residue in M^−1^cm^−1^, c is the β-LG concentration in mg/mL and P is the pathlength in cm. The secondary structure of β-LG is ascertained using the free and open-source software from Beta Structure Selection (BeStSel) [56].

### 2.8. Transmission Electron Microscopy

The JEOL JEM-2100 device was used to conduct transmission electron microscopy (TEM) at a voltage of 200 kV (JEOL Ltd., Tokyo, Japan). TEM images were obtained using an Erlangshen CCD camera (Gatan Model 782 ES500W). A few droplets of the sample solutions were deposited on a 200 mesh carbon-coated copper grid. To calculate the mean radius R_TEM_ and its standard deviation ΔR_TEM_, a total of over 100 particles were identified and utilized.

## 3. Results and Discussion

### 3.1. Formation of β-LG/CS Complexes and Effect of T80

Under stoichiometric neutrality, strong electrostatic complexation is expected to take place in polyelectrolyte/protein solutions [57]. Using Equation (9), a range of values for the mass ratio r_m_ = m_CS_/m_β-LG_ for charge neutrality was estimated [50]

(9) $$r_{m}=\frac{m_{\mathrm{CS}}}{m_{\beta -\mathrm{LG}}}=\frac{c_{\mathrm{CS}}}{c_{\beta -\mathrm{LG}}}=\frac{Z_{\beta -\mathrm{LG}}}{Z_{\mathrm{dis}}}\times \frac{M_{\mathrm{dis}}}{M_{\beta -\mathrm{LG}}}$$

**Figure 1:**
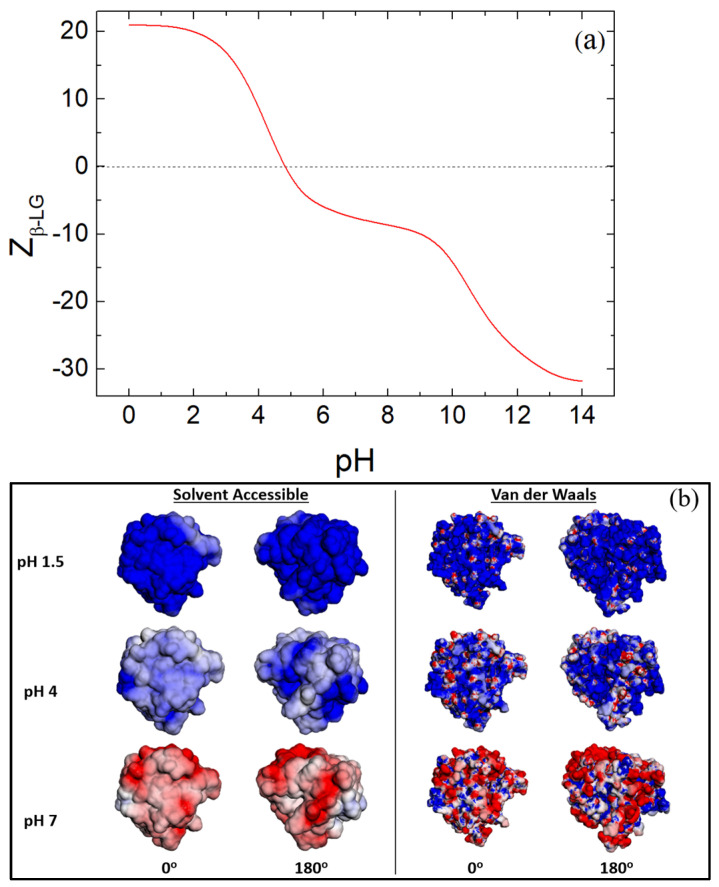
(**a**) Net charge of β-LG in relation to pH for its folded state and (**b**) electrostatic potential of β-LG at pH 1.5, 4 and 7 for the representations of solvent accessibility and van der Waals surface areas. In (b), the color scale is in the range between −5 k_B_T/e (red) and 5 k_B_T/e (blue) and the rotation of 180° is achieved on the y-axis of the protein molecule.

The molar mass of the disaccharide unit of CS, M_dis_ and of the globule of β-LG, M_β-LG_, are 379 gmol^−1^ and 18,400 gmol^−1^, respectively [50,58]. The anionic charge of one disaccharide unit of CS is Z_dis_ = 1, since the anionic behavior originates primarily from the sulfate group (pK_a_ ≈ 2.6) and not the weakly charged carboxylic group (pK_a_ ≈ 4.5) at pH 4 [59]. The isoelectric point (pI) of β-LG is 5.0 while the net charge of the protein was estimated within the range of pH 3.5 and 4.5 (Figure 1a). Therefore, the net charge of the protein was Z_β-LG_ = 8.30 ± 5.17 at pH 4 ± 0.5 and the optimum mass ratio for charge neutrality was evaluated at 0.24 ± 0.16.

The optimal complexation for polysaccharide/protein systems is achieved at the bulk charge neutrality regime, where the turbidity increases for high concentrations and a bluish tint for low concentrations can be observed, as in the present case [50,55]. These observations indicate the increase in molar mass of the formed complexes. According to Figure 1b [60], the electrostatic potential of β-LG at pH 4 is positive with small patches of negative potential. Nevertheless, electrostatic complexation between CS and β-LG is expected and any repulsions are anticipated to be minimal.

Separate β-LG/CS complexes were prepared at seven different mass ratios r_m_ at pH 4, following a geometric sequence (i.e., 0.02, 0.04, 0.08, 0.2, 0.4, 0.8 and 2). Samples with r_m_ from 0.02 to 0.2 tended to have hydrodynamic radii R_h_ larger than 1 μm (Figure S1) and a high polydispersity index (PDI), as well as more than one peak given from the CONTIN analysis. Rh values of multimodal size distributions with r_m_ from 0.02 to 0.2 were selected by the position of the dominant size distribution peak (Figure S1). These results indicate the existence of multiple populations with noted well-defined size-distribution at the nm scale. Samples with r_m_ 0.4 to 2 showed monomodal size distributions with hydrodynamic radii about 70 nm and with relatively low PDI values (0.25–0.4). For monomodal distributions, the position of the peak was chosen as the hydrodynamic radius of the system. The r_m_ value of 0.4 appeared to be the most promising given that it had the second greatest value of molar mass, M_w_, and R_g_ and R_h_ were in the order of nanometers (Figure S1). Additionally, it falls within the range of the previously calculated optimum mass ratio for charge neutrality (i.e., 0.24 ± 0.16) mentioned above.

**Figure 2:**
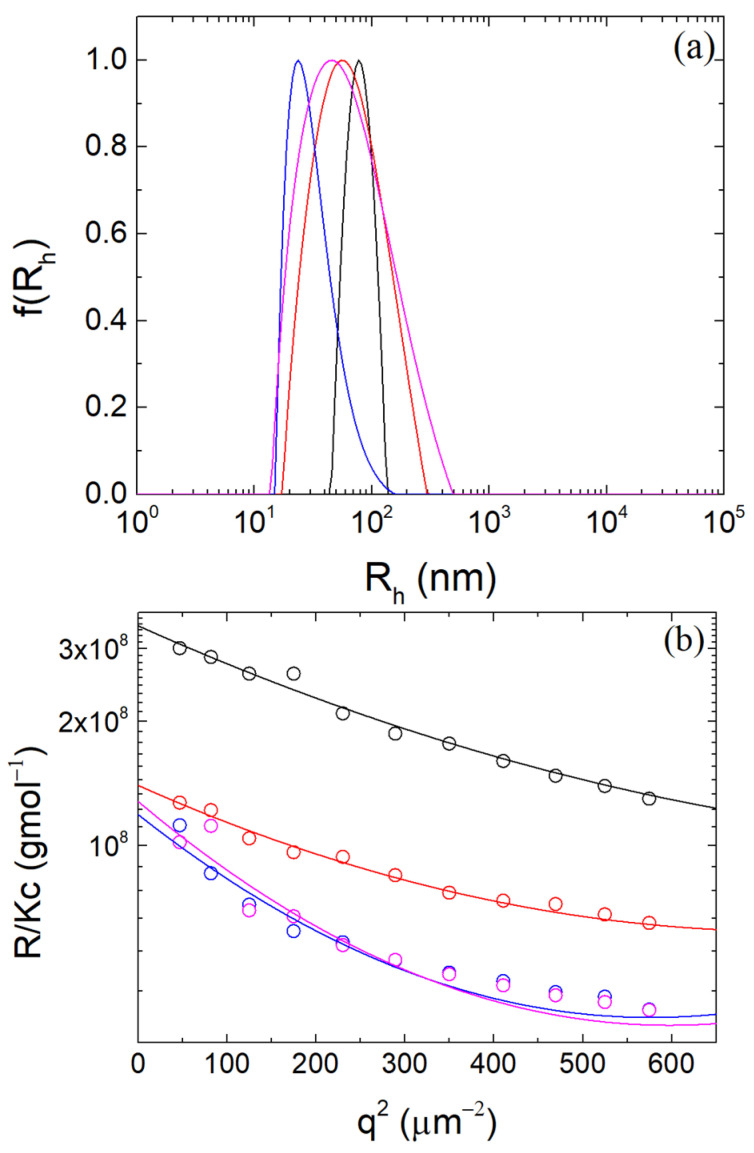
(**a**) CONTIN analysis at 90° and (**b**) Guinier plots for r_m_ values equal to 0.4 (black), 0.8 (red), 1.0 (blue) and 1.2 (magenta) at pH 4.

Samples of β-LG/CS complexes were prepared for mass ratios r_m_ in the region of the optimum r_m_ (i.e., 0.4, 0.8, 1.0 and 1.2). In Figure 2a, the sample with r_m_ 0.4 seems to be the optimal mass ratio for this system, showing the narrowest, single peak of all the four samples, the largest value of M_w_, as well as the smallest one for R_g_ in Figure 2b (Table S1). The mean hydrodynamic radius of the optimal r_m_ was 79.1 ± 3.7 nm with a molecular mass of roughly (3.45 ± 0.52)×10^8^ gmol^−1^ and a radius of gyration 86.0 ± 5.9 nm. The shape factor ρ was 1.09 ± 0.09, showing compatibility with the microgel morphology of 1.0–1.2 and close relation to the uniform spherical value of roughly 0.8 [50,61,62].

**Figure 3:**
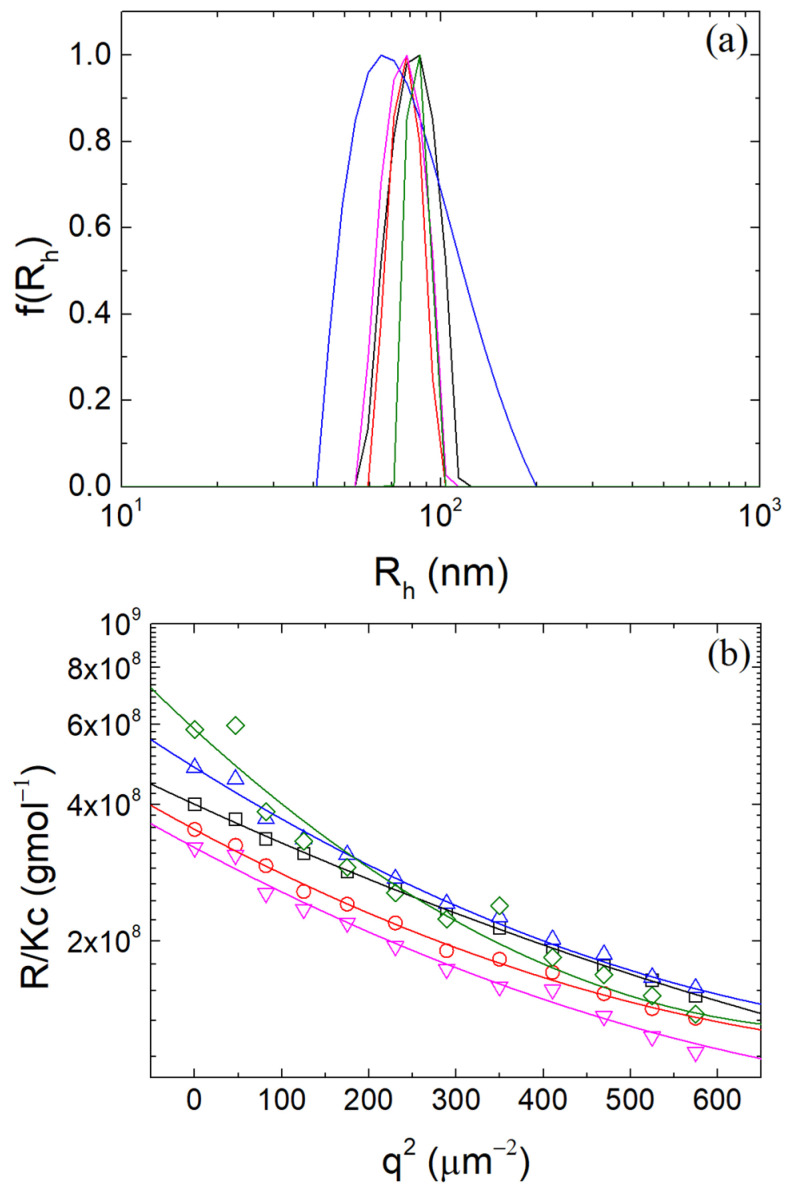
(**a**) CONTIN analysis at 90° and (**b**) Guinier plots of β-LG/CS complexes at pH 4 for 0 (black), 5 (red), 10 (blue), 20 (magenta) and 50 (olive)% T80/β-LG mass ratio.

The critical micellar concentration (CMC) of T80 is 0.014 mg/mL [36,63] and considering that the β-LG concentration in the complexes solutions was 0.1 mg/mL, the CMC of T80 translates to T80/β-LG mass ratio 14%. Five different T80/β-LG mass ratios were tested (0, 5, 10, 20 and 50%) at the optimal r_m_ of 0.4 at pH 4. It is highly expected that T80 will bind onto the protein surface and not onto the polysaccharide chains, due to its non-ionic nature and the hydrophobic regions of the protein. From Figure 3, it is observed that T80 seemed to not greatly affect the structural properties of the NPs. CONTIN analysis yielded nearly comparable peaks within the range of 80 nm. The Guinier plots in Figure 3b showed a modest increase in the M_w_ and R_g_ for contents 20 and 50%, which was possibly caused by T80’s attachment to the β-LG inside the NPs, affecting the mass of the NPs.

### 3.2. Thermal Stabilization and Morphology of β-LG/CS and β-LG/CS/T80 NPs

**Figure 4:**
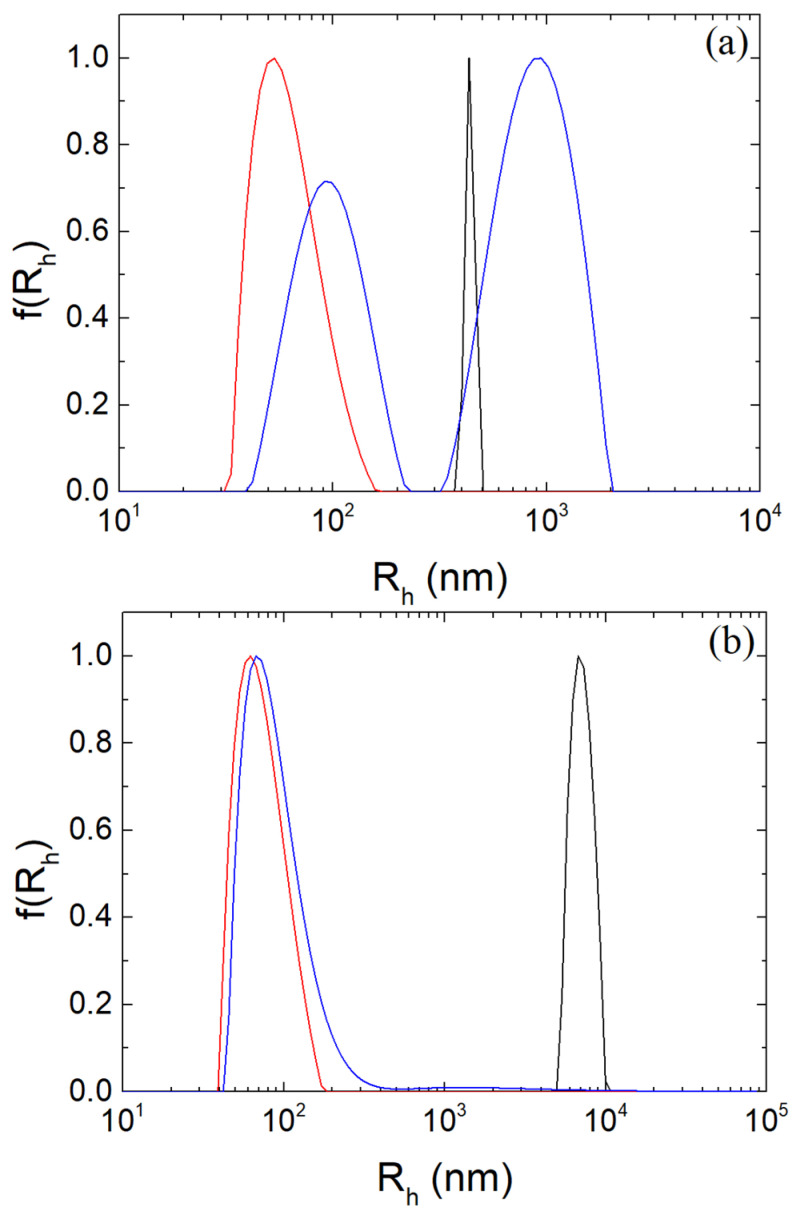
CONTIN analysis at 90° for β-LG/CS NPs at pH 1.5 (black), 4 (red) and 7 (blue): (**a**) before; (**b**) after TT.

An important property involving proteins is their thermal denaturation. Denaturation is the loss of structure of the protein. It involves the disruption and the bond breakage in the secondary, tertiary and quaternary structure of the protein. When β-LG is heated, the hydrogen bonds in its structure are affected and broken and the hydrophobic interactions are disturbed. As a result, hydrophobic sites of β-LG are exposed and protein to protein aggregation is favored upon cooling [10,11,64,65,66]. The pH values of 1.5, 4 and 7 were chosen because they are interesting for applications in human blood (drug delivery via blood stream) [67] and in organic lifeforms (drug delivery through digestion) [68], respectively. Two populations with a broad size-range are observed for untreated complexes at pH 7 in Figure 4a. The peak close to 100 nm is attributed to complexes that have survived the pH change, while the second peak around 1 μm is loose aggregates of the NPs and complexes. The SLS intensity was very low in this case, corresponding to M~10^4^ gmol^−1^. This indicated the dissociation of the electrostatic complexes and confirmed the assumptions of the destabilization NPs without TT since β-LG and CS are electrostatically repulsed due to the negative electrostatic potential of β-LG (Figure 1b) and the negative charge of the CS chains (Z_dis_ = 2 at pH 7). On the contrary, at pH 1.5, the hydrodynamic radius and the apparent molar mass increased to around 400–500 nm and by one order of magnitude, respectively. This may be attributed to NPs aggregation due to the weak charge of the sulfate and carboxylic groups of CS and the low ζ-potential as discussed in the following.

In Figure 4b, a slight increase in Rh close to 100 nm and similar population distribution to the untreated NPs at pH 4 was observed for thermally treated NPs. At pH 7, it is evident that the thermal stabilization of the NPs was achieved due to the increase in hydrophobic contacts between the protein molecules and their aggregation inside the NPs [50,55,69,70]. The mean hydrodynamic radius is 101 nm, which is slightly larger than the mean value for the NPs at pH 4 before TT, while their mass is one order of magnitude smaller (~10^7^ gmol^−1^). The radius of gyration increased to approximately 190 nm. At pH 1.5, the size of the aggregates increased by almost one order of magnitude while the size distribution was monomodal. The observed particles can be related to aggregates of β-LG/CS NPs, which is possibly due to the exposed hydrophobic areas of the protein at the surface of the NPs after its TT and the drop of surface charge of β-LG (Table S2).

**Figure 5:**
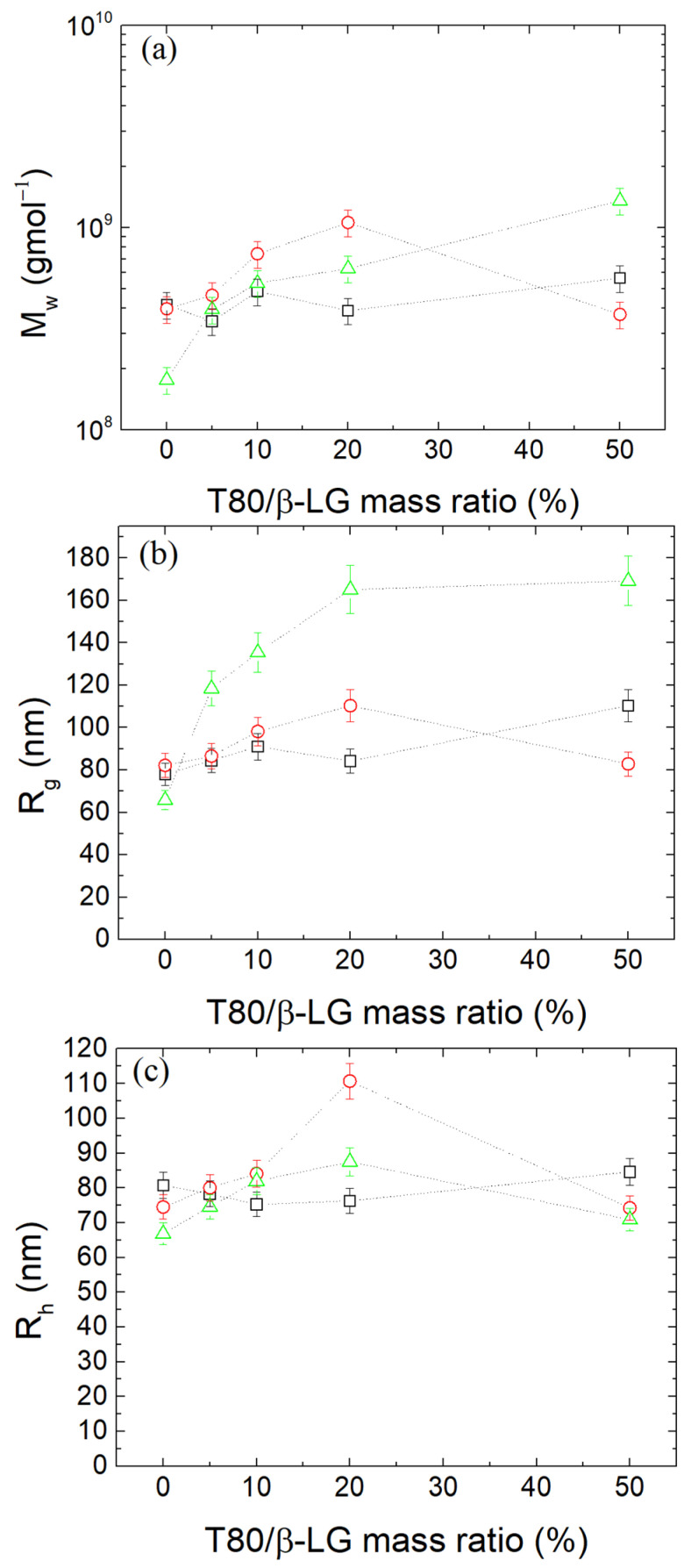
(**a**) Apparent molecular mass M_w_, (**b**) radius of gyration R_g_ and (**c**) hydrodynamic radius R_h_ (CONTIN analysis at 90°) at pH 4 for β-LG/CS/T80 NPs before (black) and after (red-T80 added after, green-T80 added before) thermal treatment.

The TT protocol seemed to occasionally fail to lead to the expected results of thermal stabilization at pH 7. Signs of failed thermal denaturation and complexes dissociating slowly in time were observed. Based on certain literature [71], it has been observed that for temperatures above 80°, the conformation of β-LG is highly reversible. Conformational changes of β-LG are important for its overall thermal aggregation. Even though the samples were thermally treated at 85^o^, either no hydrophobic areas of the protein were exposed, or the hydrophobic forces present were not strong enough to retain the structural properties of the system in such cases. This could be associated with fewer protein molecules creating aggregates with one another inside the complexes. However, based on the results on Figure 5, the thermal stabilization is primarily successful and in agreement with previous findings.

Five separate samples of β-LG/CS NPs at the optimal mass ratio r_m_ of 0.4 and at pH 4 were prepared in total with T80/β-LG mass ratios of 0, 5, 10, 20 and 50%, respectively. Samples with T80 were subjected to TT according to the mentioned thermal protocol, following two different approaches regarding the addition of T80. In the first approach, the addition of T80 was performed before TT and, in the second approach, T80 was added after TT. According to Figure 5, the addition of T80 in β-LG/CS NPs after TT slightly affects the structural properties of the NPs while the width of size distributions remained unaltered. For the case of 20% T80/β-LG mass ratio, the concentration of T80 is slightly higher than its CMC and an increase in the mass of the complexes is observed. In this case, it is assumed that self-assemblies are formed onto the outer surfaces of the NPs, causing a bridging effect between existing NPs. Generally, in organic systems of polymers with concentrations close to and slightly higher than their CMC, large and irregular aggregates, commonly referred to as animals, are formed momentarily and are usually dissolved with the increase in the concentration [72,73]. Since at a 50% mass ratio, the structural parameters return to their initial state, it is safe to assume that this bridging effect ceases to exist (Figure S3). On the contrary, the addition of T80 in β-LG/CS/T80 NPs before TT affects the overall width of the population peaks from CONTIN analysis but not the mean hydrodynamic radius. Moreover, the Guinier plots show a greater curvature, which is related to the high PDI values and relatively larger values of molecular mass. It could be supposed that in this case T80 creates domains inside the NPs, increasing the polydispersity of the NPs (Figure S4).

Since the addition of T80 before TT greatly affects the structural properties of the system, the samples were prepared and treated thermally with the presence of T80 prior to the TT to examine the extreme cases of T80 addition. It was observed that at pH 4 there was no difference before and after TT. After TT, at pH 4 and the change of pH to 7, the NPs were stabilized, while for the charge of pH to 1.5, a destabilization of the NPs after TT led to large aggregates with a narrow CONTIN population. The presence of T80 in the NPs affects the structural properties of the NPs by stabilizing the system furthermore than simply thermally treating the NPs (Figure S5).

**Figure 6:**
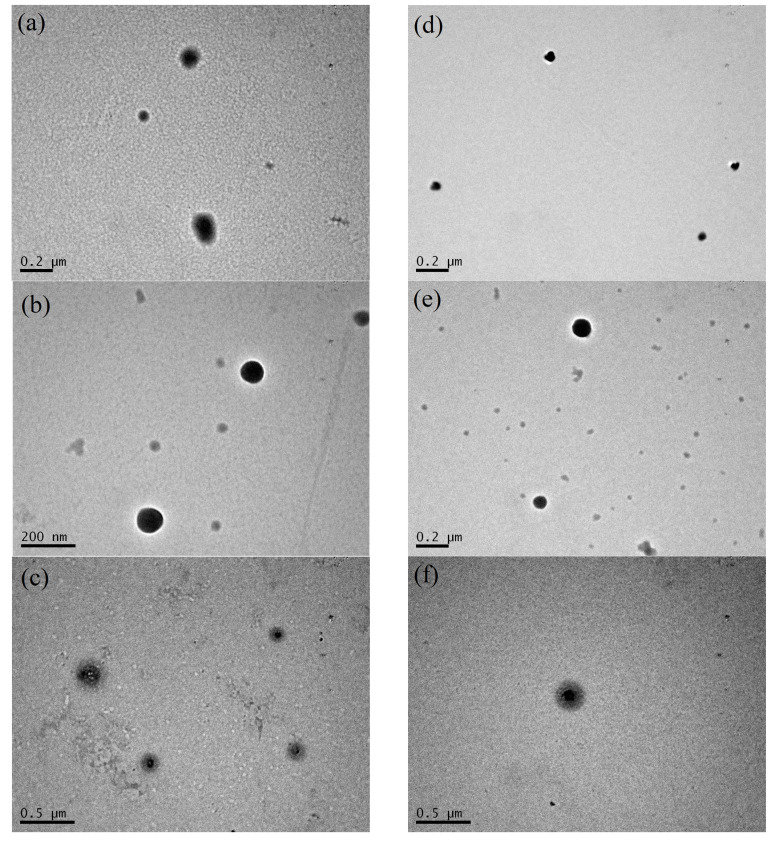
TEM images from β-LG/CS NPs with r_m_ 0.4 at 0.2 mg/mL β-LG: (**a**) before TT at pH 4; (**b**) after TT at pH 4; (**c**) after TT at pH 7. TEM images from β-LG/CS/T80 NPs at 0.2 mg/mL β-LG: (**d**) before TT at pH 4; (**e**) after TT at pH 4; (**f**) after TT at pH 7.

The shape, dimensions and morphology of the β-LG/CS and β-LG/CS/T80 NPs in Figure 6 were examined with TEM. The NPs appear to be spherical. After TT and at pH 7, they appear to have a roughly spherical external shell of a lower contrast. This shell could be related to a lower density region in comparison to the core region and could provide an explanation of the molar mass M_w_ decrease from ~10^8^ gmol^−1^ to ~10^7^ gmol^−1^ (Table S2). Apparently, the repulsion between the two biopolymers at pH 7 perturbs the integrity of the NPs at their outer periphery and not in their core. Inner and outer radius are measured for the core-shell particles. The existence of compact and core-shell NPs was reported at pH 7 after TT with and without T80, while compact NPs were observed only for pH 4 before and after TT and with and without T80. The presence of T80 seems to alter the surface of the NPs, giving them a random morphological surface while maintaining their overall spherical shape (Figure 6d). After TT at pH 4, β-LG/CS/T80 NPs seem to regain their spherical and compact morphology (Figure 6e,f). Aggregates of NPs were also found in TEM images and can be attributed to sample deposition and solvent (Figure S6). Individual particles were used for the statistical analysis of the sizes of the NPs. The estimated values of R_TEM_ ± ΔR_TEM_ for solid particles were 65 ± 17 nm for β-LG/CS NPs at pH 4 before TT, 80 ± 2 nm at pH 4 after TT and 114 ± 19 nm at pH 7 after TT. The thermally treated core-shell β-LG/CS NPs at pH 7 had an inner radius of 84 ± 8 nm and an outer radius of 179 ± 19 nm. For compact β-LG/CS/T80 NPs, the mean radius is 49 ± 3 nm at pH 4 before TT, 37 ± 1 nm at pH 4 after TT and 40 ± 2 nm at pH 7 after TT. The thermally treated core-shell β-LG/CS/T80 NPs had an inner radius of 40 ± 4 nm and an outer radius of 86 ± 10 nm. Representative size distributions from TEM images are shown in Figure S6f,g for the cases of β-LG/CS and β-LG/CS/T80, respectively. It was expected to obtain images of smaller complexes and NPs than the ones measured with DLS and SLS techniques, due to the R_h_ distributions in DLS being scattered intensity weighted and due to the sample deposition and drying of the solvent. All the discussed results agreed with the results reported from DLS/SLS measurements for NPs at optimum r_m_ = 0.4 (Figure 2, Figure S4 and S5 and Table S1).

### 3.3. Zeta Potential of β-LG/CS and β-LG/CS/T80 NPs

**Figure 7:**
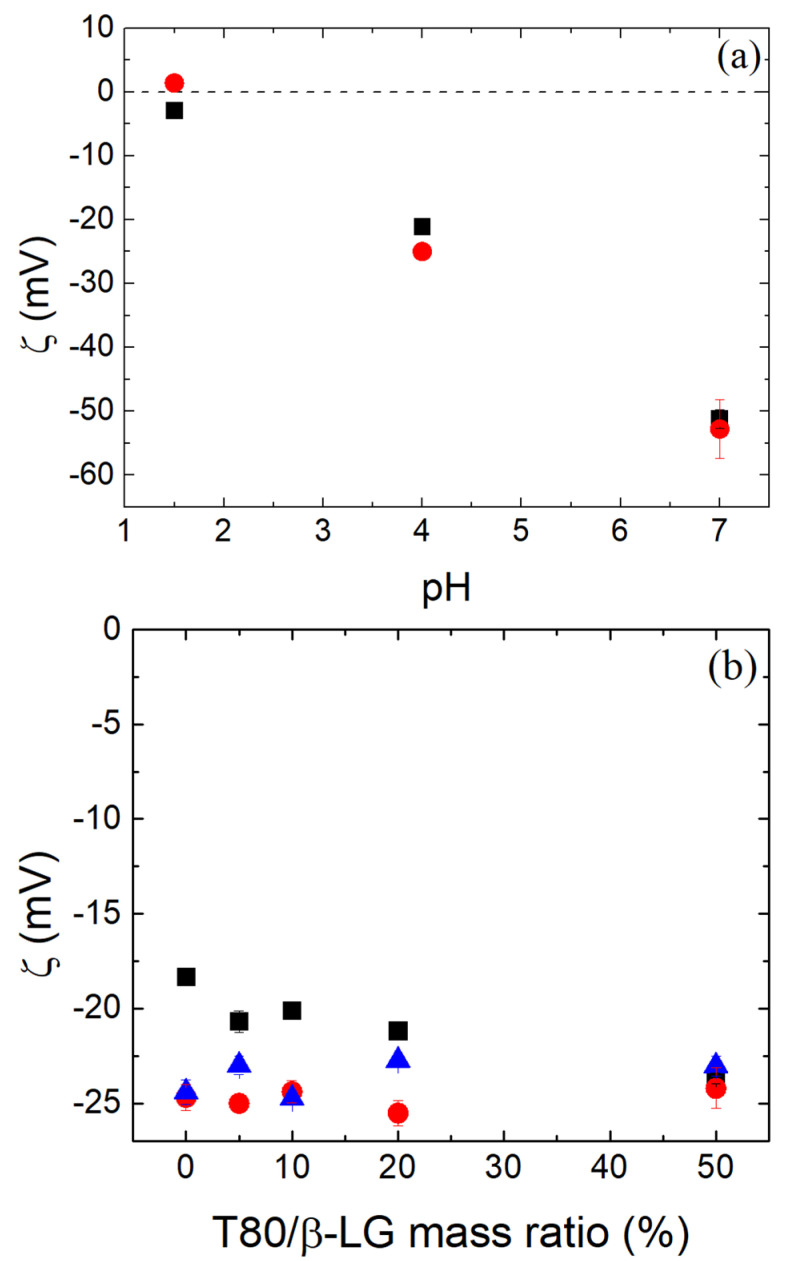
Zeta potential of β-LG/CS NPs (**a**) in relation to pH values before (black squares) and after (red circles) TT and (**b**) with the addition of different quantities of T80 at pH 4 before (black) and after TT (red circles, T80 added after TT; blue triangles, T80 added before TT).

The surface charge of the β-LG/CS NPs is important for the potential biocompatibility of the NPs and their morphological stability. Generally, for ζ-potential values close to ±30 mV, NP aggregation is limited, while positive and negative surface charges of the NPs favor the penetration and the obstruction of the cell membranes, respectively [74,75]. The surface charge of the β-LG/CS NPs at pH 4 was negative at approximately −20 mV (Figure 7a) The negative ζ-potential indicates a dominant presence of the anionic CS at the surface of the NPs and the enclosure of the overall cationic β-LG (at pH 4). At pH 7, ζ-potential was even more negative following the change of the net charge of β-LG toward negative values (Figure 1b). At pH 1.5, ζ-potential was near zero due to the loss of the anionic sulfate charge at pH < 2.6. This effect further confirms the presence of CS in the outskirts of the NPs. The addition of T80 was expected to lead to the binding of T80 on the hydrophobic regions of the protein. Due to its non-ionic nature, it was not expected to affect the overall surface charge. In Figure 7b, it is evident for the cases of thermally treated NPs that the presence of T80 does not significantly affect the surface charge.

### 3.4. β-LG Conformation in β-LG/CS and β-LG/CS/T80 NPs

**Figure 8:**
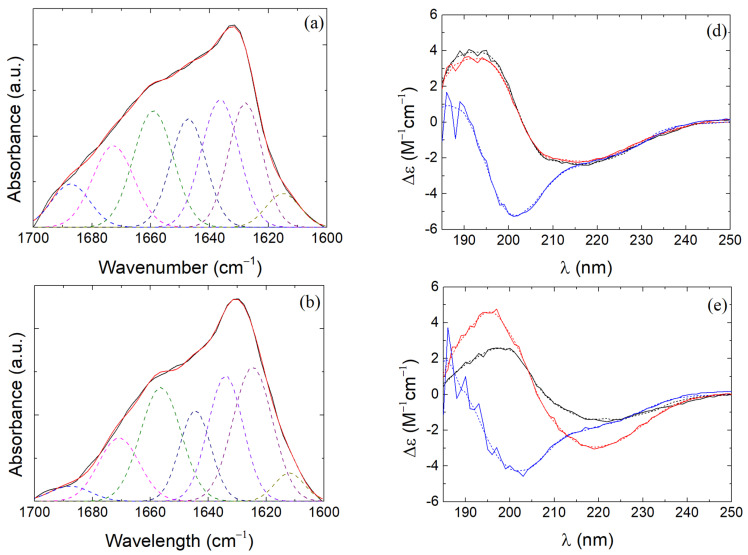

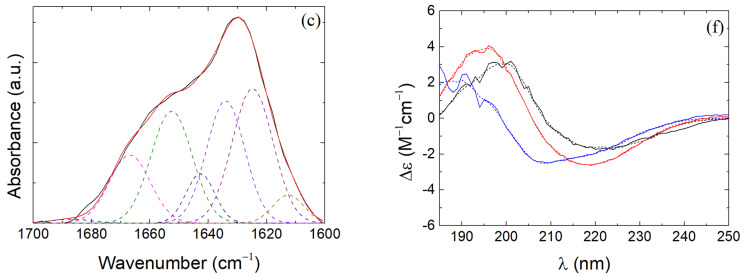
Deconvolution of the signal in the amide I region at pH 4 from: (**a**) pure β-LG before TT; (**b**) β-LG/CS NPs after TT; (**c**) β-LG/CS/T80 NPs after TT. Experimental data (solid black), fitting profile (solid red), β-sheet/β-turn (dashed blue and magenta), α-helix (dashed olive), random coil (dashed navy), extended chain (dashed violet), intramolecular β-sheet (dashed purple) and side chain moieties (dashed dark yellow). Experimental (solid lines) and fitting profiles (short dash lines) of molar ellipticity of (**d**) pure β-LG, (**e**) β-LG/CS NPs and (**f**) β-LG/CS/T80 NPs before (black) and after (red) TT at pH 4 and after TT (blue) at pH 7.

The secondary structure of β-LG from its crystal structure (by applying the DSSP method and extracting information about the hydrogen-bonded and geometrical features of the protein using a pattern-recognition process) is presented in Table 1 and concurs with previous literature [18,20]. The secondary structure of β-LG was determined by the deconvolution of the experimentally obtained amide I band spectrum (Figure 8a). The number and location of the gaussian peaks were selected based on the corresponding literature [76] and are shown in Table 2. The deconvolution of amide I signal from the FTIR spectrum of pure β-LG agrees with the results in Table 1. Therefore, the secondary structure resulting from the deconvolution of the FTIR spectra is considered reliable enough for the study of the secondary structure of β-LG in its complexes as well. In β-LG/CS complexes before and after TT (Figure 8b), the intramolecular β-sheet seems to increase while the rest of the main secondary structure elements are not affected. Similar results are reported in the cases of β-LG/CS/T80 complexes (Figure 8c), where the intramolecular β-sheet was affected and a slight decrease in β-sheet/β-turn was noted. The random coil after TT was found to decrease from 14.1% to 7.1% in the case of β-LG/CS/T80 NPs. This result could indicate the formation of hydrophobic regions that are not governed by random configurations and show an increase in the intramolecular order of the protein, which is even stronger after TT. Since intramolecular β-sheets contain more hydrophobic residues in relation to other secondary structure elements, T80 could favor these configurations [77]. It should be noted that the fitting profile on β-LG/CS/T80 NPs (Figure 8c) showed divergencies from the experimental data and that sharp difference regarding the random coil percentages could have been an overestimation.

CD was also performed for the evaluation of the secondary structure elements. The thermally untreated and pure β-LG in Table 3 agree with the results in Table 1 and Table 2 from DSSP and FTIR, respectively. The secondary structure of β-LG remains intact after TT and only the change of pH seems to affect it by increasing the irregular structure elements and the random coil segments. In the case of the untreated NPs, differences are present regarding the α-helical, β-sheet and irregular/other segments. Specifically, a-helix percentages drop from 14.6% for the pure β-LG case to 3.1% and 5.9% for the cases of β-LG/CS and β-LG/CS/T80 NPs cases, respectively. The percentages of β-sheet and β-turn remain relatively similar. The irregular/others segments showed an increase with a more dominant one for the case of β-LG/CS NPs. CD results show a strong dependence of the irregular structure of the protein on the pH of the solution but no major differences upon TT. FTIR shows a dependence of the presence of T80 in the complexes. The latter remark was seen in the CD results as well, but not as vividly as it was with the deconvolution results in Table 2. These differences in the two techniques could be attributed to the two main factors. Firstly, for FTIR measurements, it is required to dry the sample to some extent, while for CD, the secondary structure of the protein is examined in solution. Secondly, CD probes only the secondary structure of the protein, while peak shifts and intensity differences in the region of amide I band in FTIR could occur due to the presence of other groups.

### 3.5. Tryptophan Endogenous Fluorescence of Pure β-LG and in Its Complexes upon Titration of CS and T80

Tryptophan fluorescence was studied to gain insight into the electrostatic complexation between β-LG and CS and the interaction of T80 with pure β-LG and its complexes with CS. The fluorescence spectra of the tryptophan residues of β-LG were obtained through the addition in situ of CS and T80 into solutions of pure β-LG and β-LG/CS NPs. Β-LG has two tryptophan residues as follows: one in a hydrophilic environment (Trp-61) and one in a hydrophobic environment (Trp-19) (Figure S7) [78]. In the case of CS titration into pure β-LG solutions, the intensity of the tryptophan (Trp) emission peak overall increased (Figure S8). This fluorescence enhancement is closely related to the increase in hydrophobicity of the environment surrounding the tryptophan residues of β-LG and was also observed in Ottenson et al. [79]. Since CS is hydrophilic, Trp-61 is most likely affected by the addition of CS and be buried even further inside the structure of the protein. Additionally, the Trp-19 residue could also be affected in the same manner as the Trp-61 residue, but its hydrophobic environment is less likely to interact with CS.

The titration of T80 into pure β-LG solutions led to the reduction in the maximum intensity of the fluorescence spectra of the tryptophan residues (Figure S9a). This fluorescence quenching indicates the successful binding of T80 onto the tryptophan residues and their surrounding regions. The quenching constant (K_SV_) of T80 onto β-LG was quantified by the Stem–Volmer equation [80,81], $\frac{F_{o}-F}{F}=K_{\mathrm{SV}}\times c_{T80}$, where K_SV_ is extracted from the slope of the linear fit of the experimental ratio $\frac{F_{o}-F}{F}$ in relation to c_T80_ (Figure S9b), and F_o_ is the maximum intensity without T80. Moreover, the binding constant (K_A_) and the number of binding sites n per globule are extracted from the intercept and the slope of the linear fit of $\ln\left[\frac{F_{o}-F}{F}\right]$ in relation to $\ln\left[c_{T80}\right]$ under the relation $\ln\left[\frac{F_{o}-F}{F}\right]={\mathrm{lnK}}_{A}+n\times \ln\left[c_{T80}\right]$ (Figure S9c) [81,82]. In this case, the calculated values of the constants are $K_{\mathrm{SV}}\approx 0.2\times 10^{4}$ M^−1^, $K_{A}\approx 5\times 10^{4}$ M^−1^ and the number of binding sites is equal to n≈1.3. Prior research on human serum albumin revealed that T80 binding was moderate, with binding constants in the range of roughly 10^3^ M^−1^, with one to three molecules of surfactant bound to the albumin [83,84]. These results are comparable to the present case of β-LG of 10^3^–10^4^ M^−1^.

T80 binding was also observed for the β-LG/CS complexes and NPs (Figure S10). The quenching constant was found $0.4\times 10^{4}$ M^−1^ approximately, the binding constant was $0.5\times 10^{4}$ M^−1^ and the number of binding sites n decreased to 1.0 (Table 4). The difference of one order of magnitude between the two binding constants could be attributed to the fact that β-LG is mostly hidden inside its complexes with CS and T80 finds it difficult to bind onto the protein sites. Since only the FL tryptophan spectra of β-LG were investigated, the expected number of binding sites is between 1 and 2 and the results indicate the binding of T80 onto one tryptophan residue, most likely Trp-19, and its ambient surroundings.

### 3.6. Hydrophobicity of β-LG/CS and β-LG/CS/T80 NPs

The surface of β-LG contains hydrophobic residues (Figure S7), which makes the prepared NPs potential nanocarriers of hydrophobic compounds. For pyrene experiments, the hydrophobicity was determined from the ratio of the intensity I_1_ of the first peak (372 nm) over the intensity I_3_ of the third peak (383 nm). Values close to 1.9 indicate a hydrophilic environment, while values close to 1.0 indicate a hydrophobic environment [85,86]. Solutions of pure β-LG show a relatively polar environment with I_1_/I_3_ at 1.60 despite its hydrophobic patches (Table 5). When β-LG was thermally treated, hydrophobicity increased slightly with I_1_/I_3_ at 1.54. With the addition of T80, I_1_/I_3_ acquired the value of 1.27 before and after TT, showing that T80 increased the hydrophobic regions that may accommodate pyrene. NPs without T80 were relatively hydrophobic with values of I_1_/I_3_ at 1.36 and 1.30 before and after TT, respectively. These results are expected because of the enclosure of β-LG inside its complexes with CS and the hydrophobic regions and patches of the protein. Additionally, due to the complexation of β-LG and CS, hydrophobic domains inside the NPs are anticipated to form. The addition of T80 seemed not to affect the overall hydrophobicity (1.32 and 1.31, respectively) which indicates that T80 binds entirely onto the protein. This could be explained by the hydrophilicity of CS and the non-ionic nature of T80, which leads to the assumption that T80 does not bind onto CS and even on the surface of the NPs it is more likely to interact with β-LG patches.

Fluorescence experiments with ANS as a probe (Figures S11 and S12) were performed to quantify the surface hydrophobicity S_o_ of the NPs. Surface hydrophobicity was determined from the slope of the fluorescence intensity at maximum of the ANS emission spectrum in relation to the concentration of β-LG in the NPs, since the intensity is expected to increase by increasing the β-LG concentration [87]. Pure β-LG has a more hydrophobic surface (S_o_ = 144× 10^5^ CPS·mL/mg) than the one of the β-LG/CS and β-LG/CS/T80 NPs (S_o_ = 28 × 10^5^ CPS·mL/mg and S_o_ = 127× 10^5^ CPS·mL/mg, respectively) verifying the dominant presence of CS on the surface of the NPs (Table 5). Upon TT, the NPs (S_o_ = 334× 10^5^ CPS·mL/mg) became more hydrophobic, indicating the exposure of hydrophobic sites of the protein. The addition of T80 increased the surface hydrophobicity, which means that T80 binds on inner surfaces of the NPs and secures hydrophobic surface patches of the protein. The term “inner surfaces” refers to the microgel morphology of the NPs where hydrophilic and hydrophobic regions are present inside the NPs. These are also the regions where substances can be encapsulated (both hydrophobic or hydrophilic) or where water molecules can be absorbed inside the NPs, causing them to swell. Based on the pyrene results, the overall hydrophobicity of the β-LG/CS and the β-LG/CS/T80 is similar for both cases, indicating that T80 micelles are neither formed nor do they exist freely inside the solution. However, the increase in surface hydrophobicity based on the ANS results indicates the formation of self-assemblies of T80 inside the NPs, which in turn create even more hydrophobic regions onto the inner and outer surfaces of the NPs.

### 3.7. Stability of β-LG/CS and β-LG/CS/T80 NPs in Salt and Biological Fluids and over Time

**Figure 9:**
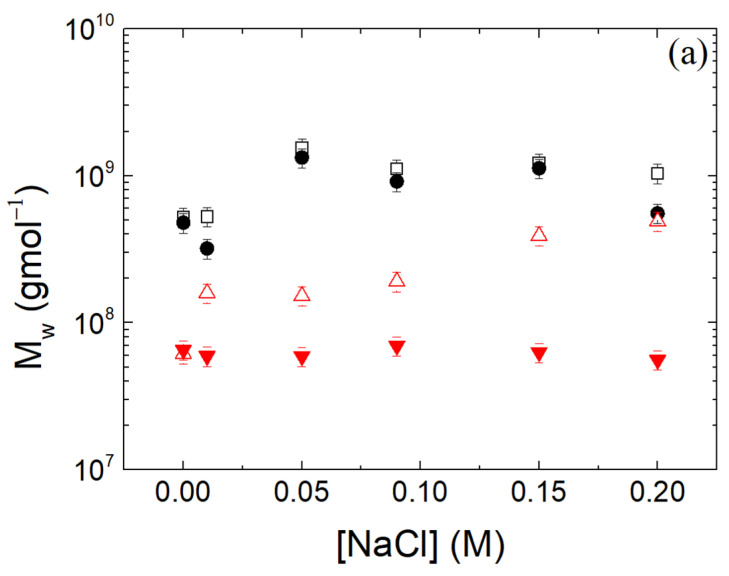

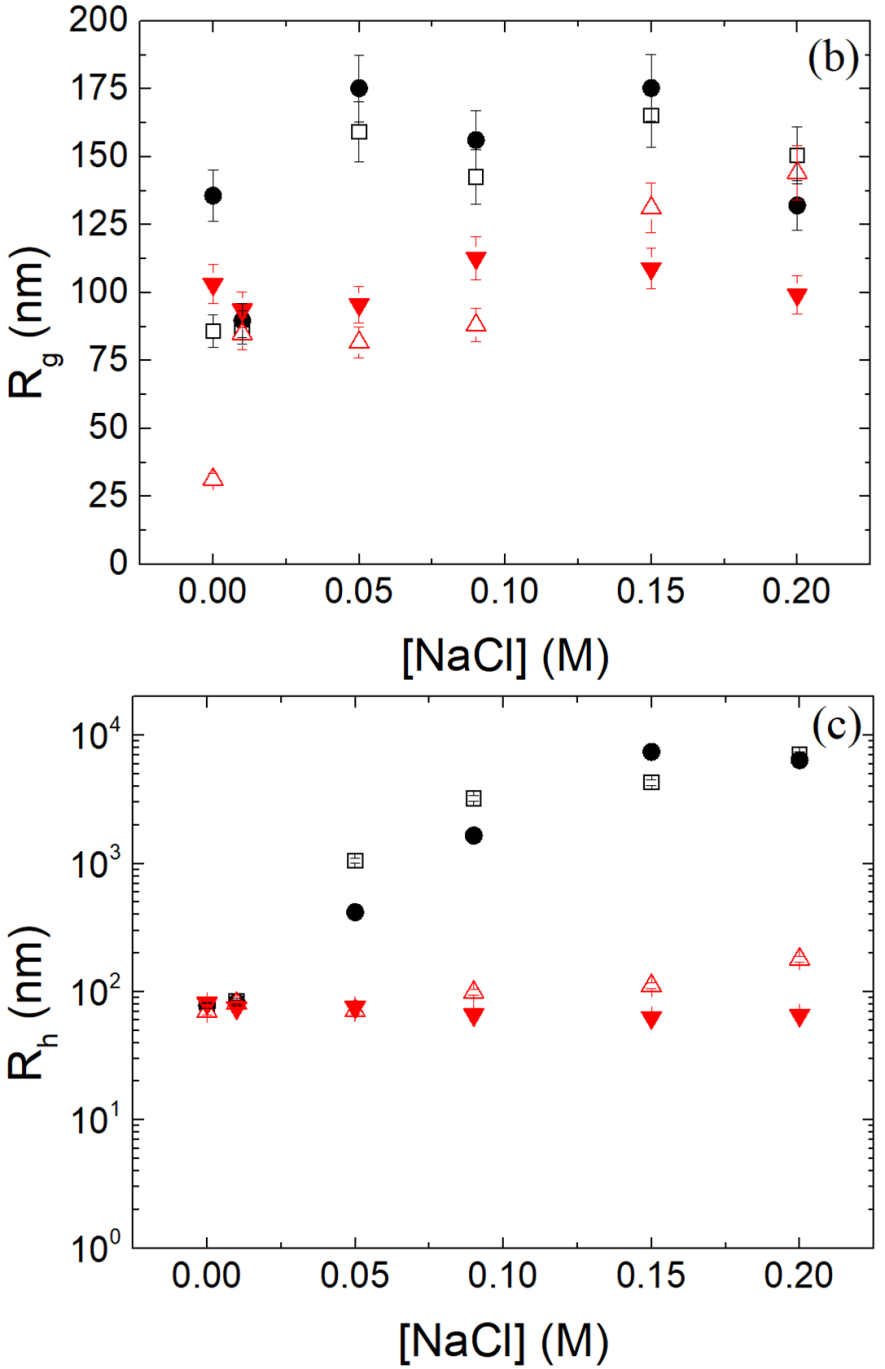
(**a**) Molecular mass M_w_, (**b**) radius of gyration R_g_ and (**c**) hydrodynamic radius R_h_ (CONTIN 90°) in relation to NaCl concentration for thermally treated β-LG/CS NPs with (filled circles and reverse triangles) and without (unfilled squares and triangles) T80 at pH 4 (black) and 7 (red).

NPs underwent aggregation by increasing the salt content at pH 4 (Figure 9). The molar masses and the hydrodynamic increased to ~10^9^ gmol^−1^ and ~10^4^ nm, respectively, as the salt concentration increased, while the radii of gyration exhibited a slight increase as well. These results were expected due to the screening of the electrostatic repulsions between the NPs. On the contrary, at pH 7, the NPs remained stable for the cases of T80 being present or not. The absence of T80 at pH 7 also leads to interparticle aggregation with a slower rate, while its present fortifies the stability of the NPs against the electrostatic interactions screening (Figure 9).

A reference FBS:PBS mixture with H_2_O was examined to record aggregates of FBS in the solution, since the latter is a mixture of proteins itself (Figure S13). The size distribution peaks marked the existence of β-LG/CS NPs in the biological fluids. For NPs without T80, one single peak is reported at approximately 100 nm and is attributed to stable NPs in the solution, while for NPs with T80, a bimodal distribution is taking place with one peak around the 80–100 nm regime. A few other populations were also observed at about 10 and 60 nm by LS measurements and are possibly attributed to the aggregates or complexes of the FBS solution itself, similarly to the results of the reference sample (Figure S13a). The Guinier plots indicate well-defined NPs and a notable difference in the scattering intensity between the FBS complexes and the studied NPs (Figure S13b). The molar masses of the NPs were approximately $3\times 10^{8}$ gmol^−1^ (Table S3), like previous results (Table S1). The reported hydrodynamic radii were around 20 nm with high PDI values (~0.5) and dubious shape factors due to the presence of FBS aggregates. Based solely on the size distribution and the reported molar masses, as well as the overall Guinier plots, it is assumed that the NPs show stability to a certain degree in FBS:PBS solutions and, therefore, the results show their biocompatibility to biological fluids with high salinity.

The stability of β-LG/CS and β-LG/CS/T80 NPs with r_m_ = 0.4 at pH 4 before and after TT was tested for the duration of 20 or 30 days. NPs prepared at the optimal mass ratio r_m_ 0.4 showed no change to their molar mass, hydrodynamic radius or shape factor, but did show a slightly neglectable increase to their radius of gyration, reinforcing the choice of the optimal mass ratio r_m_ (Table S4). Thermally treated β-LG/CS NPs exhibited stability over time at pH 1.5, 4 and 7, respectively. In the case of pH 1.5, NP aggregates seem to slowly dissolve and shrink as observed by the decrease in their molar mass and radius of gyration and the slight decrease in the hydrodynamic radius, respectively (Table S5). Based solely on SLS measurements, β-LG/CS/T80 NPs before and after TT at pH 4 remained stable after the span of 30 days, particularly for those not thermally treated (Table S6). Overall, the structural parameters revealed by LS measurements imply NP stability over time, since they show little or no change.

## 4. Conclusions

In this research study, the electrostatic complexation of β-LG with the negatively charged CS was applied for the formation of well-defined complexes NPs at pH 4 and r_m_ = 0.4 and thermal stabilization against disintegration at neutral pH was achieved. Additionally, the NPs were modified by the addition of the non-ionic T80. T80 addition after thermal treatment caused bridging between the NPs for T80 concentration close to its CMC, which did not take place at a higher T80 concentration. Thermal stabilization in the presence of T80 achieved narrower size distributions upon the change from acidic to neutral pH and retained their microgel morphology with little to no changes of their shape factor. NP aggregation was recorded at pH 1.5 before and after thermal treatment. The surface charge of the NPs was negative and T80 had no significant effect on it. The secondary structure of β-LG had small differences in thermally treated complexes and a strong dependence on pH. Fluorescence enhancement was reported upon addition of CS in pure β-LG solutions, while fluorescence quenching was reported upon titration of T80, indicating its successful binding onto the protein molecules. The hydrophobicity of the NPs was relatively high even without the addition of T80, but the relative surface hydrophobicity showed a robust reliance in relation to T80 concentration. The increase in surface hydrophobicity with the addition of T80 is likely related to self-assemblies onto the inner and outer surfaces of the NPs. Thermally treated NPs with T80 at pH 7 showed remarkable stability upon the increase in ionic strength and upon their injection into biological FBS:PBS fluids. The colloidal properties of the NPs showed little to no changes within the span of 1 month, particularly for the β-LG/CS/T80 NPs. All the above remarks indicate the potential suitability of the NPs for encapsulation and delivery of hydrophobic compounds for food science and pharmaceutical applications.

## Figures and Tables

**Table 1:** Calculated secondary structure elements for β-LG from X-ray crystallography data.

Composition of Secondary Structure Elements	Three State Composition (%)	Analysis vs. DSSP	DSSP
Helix	16.04	Similarity%	100
Sheet	40.74	Matthews CC	1
Other	43.20		

**Table 2:** Secondary structure of β-LG determined from ATR-FTIR data. β-LG at 0.1 mg/mL. In β-LG/CS/T80 complexes, T80 at 50% T80/β-LG mass ratio and at pH 4 (r_m_ = 0.4) (error in estimation of percentages is approximately ±1.0%).

Assignment	Β-Sheet/Β-Turn	A-Helix	Random Coil	Intramolecular β-Sheet	Others
Wavenumbers (cm^−1^)	1685–1663	1655–1650	1648–1644	1632–1621	1639–1635 1616–1600
Pure β-LG	21.3%	19.6%	16.2%	17.8%	25.1%
β-LG/CS (NoTT)	15.4%	22.0%	13.9%	20.3%	28.5%
β-LG/CS (TT)	15.1%	21.8%	13.3%	25.0%	24.7%
β-LG/CS/T80 (NoTT)	18.5%	22.2%	14.1%	19.7%	25.5%
β-LG/CS/T80 (TT)	14.8%	23.0%	7.1%	27.3%	27.8%

**Table 3:** Secondary structure estimated by CD. Content of pure β-LG and β-LG in β-LG/CS and β-LG/CS/T80 NPs before and after TT at pH 4 and after TT at pH 7 (error in estimation of percentages is approximately ±1.0%).

Pure β-LG	A-Helix	Β-Sheet	Β-Turn	Irregular/Others
Untreated/pH 4	14.6%	35.0%	10.3%	40.1%
Treated/pH 4	13.1%	35.4%	10.9%	40.7%
Treated/pH 7	13.2%	22.3%	14.6%	49.8%
**β-LG/CS NPs**	**α-helix**	**β-sheet**	**β-turn**	**Irregular/Others**
Untreated/pH 4	3.1%	36.4%	15.1%	45.3%
Treated/pH 4	9.8%	30.0%	10.7%	49.6%
Treated/pH 7	9.2%	28.0%	17.5%	45.3%
**β-LG/CS/T80 NPs**	**α-helix**	**β-sheet**	**β-turn**	**Irregular/Others**
Untreated/pH 4	5.9%	37.4%	13.5%	43.2%
Treated/pH 4	10.4%	33.6%	12.4%	43.4%
Treated/pH 7	16.1%	26.7%	13.5%	43.7%

**Table 4:** Quenching constant K_SV_, binding constant K_A_ and number of binding sites n per protein globule of T80 from tryptophan fluorescence.

Parameter/Sample	Pure β-LG	β-LG/CS NPs
K_SV_ (10^4^ M^−1^)	0.211 ± 0.032	0.383 ± 0.104
K_A_ (10^4^ M^−1^)	4.96 ± 0.64	0.451 ± 0.586
n	1.32 ± 0.20	1.00 ± 0.27

**Table 5:** Results from pyrene fluorescence experiments and surface hydrophobicity on pure β-LG, pure β-LG and T80, β-LG/CS NPs and β-LG/CS/T80 NPs before and after TT (r_m_ = 0.4, pH = 4 and T80/β-LG mass ratio = 50%).

State	Sample/Parameter	I_1_/I_3_	S_o_ (10^5^ CPS·mL/mg)
Before TT	Pure β-LG	1.60 ± 0.08	144 ± 9
	Pure β-LG/T80	1.27 ± 0.07	454 ± 20
	β-LG/CS NPs	1.36 ± 0.07	28.0 ± 0.9
	β-LG/CS/T80 NPs	1.32 ± 0.07	127 ± 7
After TT	Pure β-LG	1.54 ± 0.08	128 ± 19
	Pure β-LG/T80	1.34 ± 0.07	414 ± 40
	β-LG/CS NPs	1.30 ± 0.07	334 ± 9
	β-LG/CS/T80 NPs	1.31 ± 0.07	422 ± 33

## Data Availability

Data is contained within the article or Supplementary Materials.

## Supplementary Materials

The following supporting information can be downloaded at: https://www.mdpi.com/article/10.3390/polym16141995/s1, Figure S1: (a) Molecular mass M_w_, (b) radius of gyration R_g_ and (c) hydrodynamic radius R_h_ (CONTIN at 90°) in relation to seven different values of mass ratio r_m_, i.e. 0.02, 0.04, 0.08, 0.2, 0.4, 0.8 and 2, at pH 4; Table S1: Experimental results of β-LG/CS NPs for r_m_ values of 0.4, 0.8, 1.0 and 1.2 at pH 4 (Uncertainties: δM_w_ = ±15%, δR_g_ = ±7%, δR_h_ = ±5% and δρ = ±8%); Figure S2. Guinier plots for β-LG/CS NPs before (open points) and after (closed points) thermal treatment at pH 1.5 (black), 4 (red) and 7 (blue); Table S2. Experimental results of thermally treated and not β-LG/CS NPs for r_m_ 0.4 at pH 1.5, 4 and 7 (Uncertainties: δM_w_ = ±15%, δR_g_ = ±7%, δR_h_ = ±5% and δρ = ±8%); Figure S3. (a) CONTIN analysis at 90^o^ and (b) Guinier plots of β-LG/CS complexes after TT at pH 4 for 0 (black), 5 (red), 10 (blue), 20 (magenta) and 50 (olive)% T80/β-LG mass ratios with T80 being added after TT; Figure S4. (a) CONTIN analysis at 90^o^ and (b) Guinier plots of β-LG/CS complexes after TT at pH 4 for 0 (black), 5 (red), 10 (blue), 20 (magenta) and 50 (olive)% T80/β-LG mass ratios with T80 being added before TT; Figure S5: (a) CONTIN analysis at 90° and (b) Guinier plots of β-LG/CS complexes for 50% T80/β-LG mass ratio at pH 1.5 (black), 4 (red) and 7 (blue), before (dashed-dotted/dashed lines) and after (solid lines) TT (T80 added before TT); Figure S6. TEM images from clusters and aggregates of β-LG/CS NPs at 0.2 mg/mL β-LG (a) after TT at pH 4 and (b) at pH 7. TEM images from clusters and aggregates of β-LG/CS/T80 NPs at 0.2 mg/mL β-LG (c) before TT and (d) after TT at pH 4 and (e) after TT at pH 7. Size distributions of β-LG/CS NPs at pH 4 after TT (f) without and g) with T80 extracted from multiple TEM images (Gaussian fits are presented along the respected size distributions); Figure S7. (a) Ratio of non-polar to polar sites of β-LG molecule at 0° and 180°. The ratio of non-polar/polar residues color scale is in the range between 0.6 (purple) and 2.3 (green). (b) Positions of Trp 19 and Trp 61 in plan view of the β-LG molecule respectively; Figure S8. (a) Tryptophan fluorescence of β-LG with the addition in situ of CS in pure β-LG at pH 4 with r_m_ values of 0.0 (black), 0.1 (red), 0.2 (blue), 0.3 (magenta), 0.4 (green), 0.5 (navy blue), 0.6 (purple), 0.7 (wine), 0.8 (brown), 0.9 (olive) and 1.0 (cyan). (b) Maximum peak intensities in relation to r_m_ values for the case of the addition in situ of CS in pure β-LG at pH 4 (Maximum peaks at 328 nm were selected after smoothing the experimental fluorescence spectra); Figure S9. (a) Tryptophan fluorescence of β-LG with the addition in situ of T80 in pure β-LG at pH 4 with r_m_ values of 0 (black), 5 (red), 10 (blue), 15 (magenta), 20 (green), 25 (navy blue), 30 (violet), 35 (purple), 40 (wine), 45 (dark yellow), 50 (dark blue), 55 (cyan) and 60 (brown). Linear fits of the fluorescence intensity at maximum from titration of T80 at pH 4 for the evaluation of (b) the quenching constant K_SV_ and c) the binding constant K_A_ and the number of binding sites n per protein globule (Maximum peaks at 328 nm were selected after smoothing the experimental fluorescence spectra); Figure S10. (a) Tryptophan fluorescence of β-LG with the addition in situ of T80 in β-LG/CS NPs at pH 4 with r_m_ values of 0 (black), 5 (red), 10 (blue), 15 (magenta), 20 (green), 25 (navy blue), 30 (violet), 35 (purple), 40 (wine), 45 (dark yellow), 50 (dark blue), 55 (cyan) and 60 (brown). Linear fits of the fluorescence intensity at maximum from titration of T80 at pH 4 for the evaluation of (b) the quenching constant K_SV_ and (c) the binding constant K_A_ and the number of binding sites n per protein globule (the last four points from the graphs are excluded from the linear fit due to intensity saturation) (Maximum peaks at 328 nm were selected after smoothing the experimental fluorescence spectra); Figure S11. ANS fluorescence spectra of pure β-LG (a,b), pure β-LG/T80 (c,d), β-LG/CS (e,f), and β-LG/CS/T80 (g,h) NPs before TT. C_β-LG_: 0.1 (black), 0.08 (red), 0.06 (blue) and 0.04 (magenta) mgmL^-1^ (Maximum peaks at 496-505 nm were selected after smoothing the experimental fluorescence spectra); Figure S12. ANS fluorescence spectra of pure β-LG (a,b), pure β-LG/T80 (c,d), β-LG/CS (e,f), and β-LG/CS/T80 (g,h) NPs after TT. C_β-LG_: 0.1 (black), 0.08 (red), 0.06 (blue) and 0.04 (magenta) mgmL^−1^ (Maximum peaks at 496-505 nm were selected after smoothing the experimental fluorescence spectra); Figure S13. (a) CONTIN analysis at 90° and (b) Guinier plots for a reference sample with H_2_O (black) an thermally treated β-LG/CS complexes at pH 7 with (blue) and without (red) T80 in the FBS:PBS solutions; Table S3. Experimental results of a reference sample with H_2_O and thermally treated β-LG/CS and β-LG/CS/T80 NPs in FBS:PBS solution at pH 7 (Uncertainties: δM_w_ = ±15%, δR_g_ = ±7%, δR_h_ = ±5% and δρ = ±8%); Table S4. Molecular mass M_w_, radius of gyration R_g_, hydrodynamic radius R_h_ from CONTIN analysis in 90^o^ and shape factor ρ of β-LG/CS NPs at pH 4 with r_m_ 0.4 and 0.8 for day 0, 15 and 30 (Uncertainties: δM_w_ = ±15%, δR_g_ = ±7%, δR_h_ = ±5% and δρ = ±8%); Table S5. Molecular mass M_w_, radius of gyration R_g_, hydrodynamic radius R_h_ from CONTIN analysis in 90^o^ and shape factor ρ of thermally treated β-LG/CS NPs with r_m_ 0.4 and at pH 1.5, 4 and 7 for day 0 and 20 (Uncertainties: δM_w_ = ±15%, δR_g_ = ±7%, δR_h_ = ±5% and δρ = ±8%); Table S6. Molecular mass M_w_, radius of gyration R_g_, hydrodynamic radius R_h_ from CONTIN analysis in 90° and shape factor ρ of thermally treated (TT) and not (NoTT) β-LG/CS/T80 NPs (50% T80/β-LG mass ratio) with r_m_ 0.4 and at pH 4 for day 0 and 30 (Uncertainties: δM_w_ = ±15%, δR_g_ = ±7%, δR_h_ = ±5% and δρ = ±8%).
