# Supplementary material for: Biocompatible Preparation of Beta-Lactoglobulin/Chondroitin Sulfate Carrier Nanoparticles and Modification of Their Colloidal and Hydropathic Properties by Tween 80
Source: Polymers (Basel). 2024 Jul 12;16(14):1995. doi: 10.3390/polym16141995 (PMC11280915; doi:10.3390/polym16141995)
Supplement: Supplementary file 1 [file polymers-16-01995-s001.zip › polymers-3098987-supplementary.pdf]

polymers

Supporting Information

Biocompatible Preparation of Beta-Lactoglobulin/Chondroitin Sulfate Carrier Nanoparticles and Modification of Their Colloidal and Hydropathic Properties by Tween 80

Ioannis Pispas [1], Nikolaos Spiliopoulos [2] and Aristeidis Papagiannopoulos [1,*]

[1] Theoretical and Physical Chemistry Institute, National Hellenic Research Foundation, 48 Vassileos Constantinou Avenue, 11635 Athens, Greece; johnpispas@gmail.com
[2] Department of Physics, University of Patras, 26504 Patras, Greece; nspiliop@physics.upatras.gr
* Correspondence: apapagiannopoulos@eie.gr

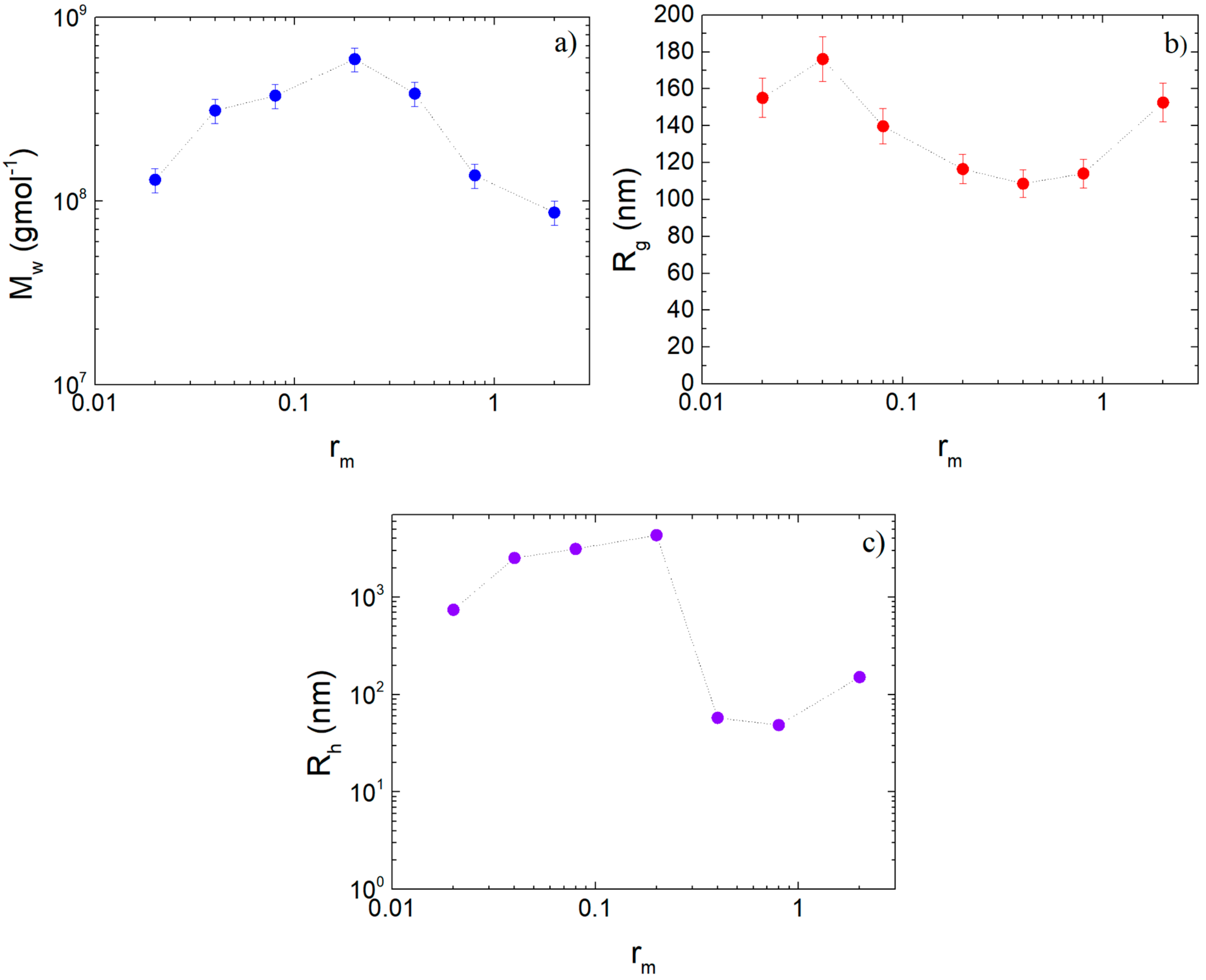

Figure S1. (**a**) Molecular mass M_w, (**b**) radius of gyration R_g and (**c**) hydrodynamic radius R_h (CONTIN at 90°) in relation to seven different values of mass ratio r_m, i.e. 0.02, 0.04, 0.08, 0.2, 0.4, 0.8 and 2, at pH 4.

Table S1. Experimental results of β-LG/CS NPs for r_m values of 0.4, 0.8, 1.0 and 1.2 at pH 4 (Uncertainties: δM_w=±15%, δR_g=±7%, δR_h=±5% and δϱ=±8%).

	SLS		DLS (Cumulant, 90°)		DLS (CONTIN, 90°)	
r_m	M_w (gmol^{-1})	R_g (nm)	R_h (nm)	PDI	R_h (nm)	ϱ
0.4	$3.39\cdot10^{8}$	83.1	65.8	0.257	76.8	1.26
0.8	$1.40\cdot10^{8}$	81.5	49.8	0.394	62.5	1.64
1.0	$1.23\cdot10^{8}$	114	30.5	0.495	49.2	3.75
1.2	$1.13\cdot10^{8}$	103	43.9	0.471	59.7	2.34

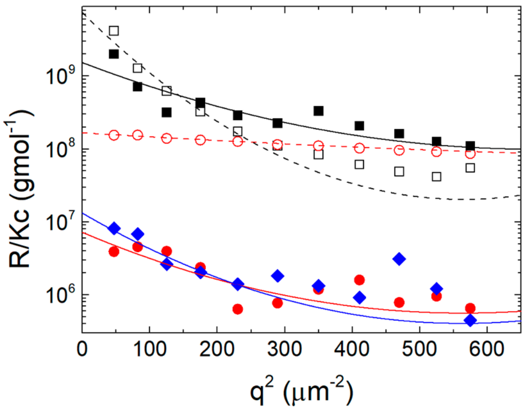

Figure S2. Guinier plots for β-LG/CS NPs before (open points) and after (closed points) thermal treatment at pH 1.5 (black), 4 (red) and 7 (blue).

Table S2. Experimental results of thermally treated and not β-LG/CS NPs for r_m 0.4 at pH 1.5, 4 and 7 (Uncertainties: δM_w=±15%, δR_g=±7%, δR_h=±5% and δϱ=±8%).

	SLS		DLS (Cumulant, 90°)		DLS (CONTIN, 90°)	
State - pH	M_w (gmol^{-1})	R_g (nm)	R_h (nm)	PDI	R_h (nm)	ϱ
NoTT - 1.5	$7.39\cdot10^{9}$	250.3	465	0.101	450	0.54
TT - 1.5	$1.53\cdot10^{9}$	156	7750	0.349	7000	0.02
NoTT – 4	$1.67\cdot10^{8}$	62.8	63.7	0.211	60.3	0.99
TT - 4	$7.19\cdot10^{8}$	165	71.4	0.100	69.4	2.30
NoTT – 7	-	-	180	0.538	827 (57%)<br>84 (33%)	-
TT - 7	$1.32\cdot10^{7}$	193	84.4	0.256	101	1.91

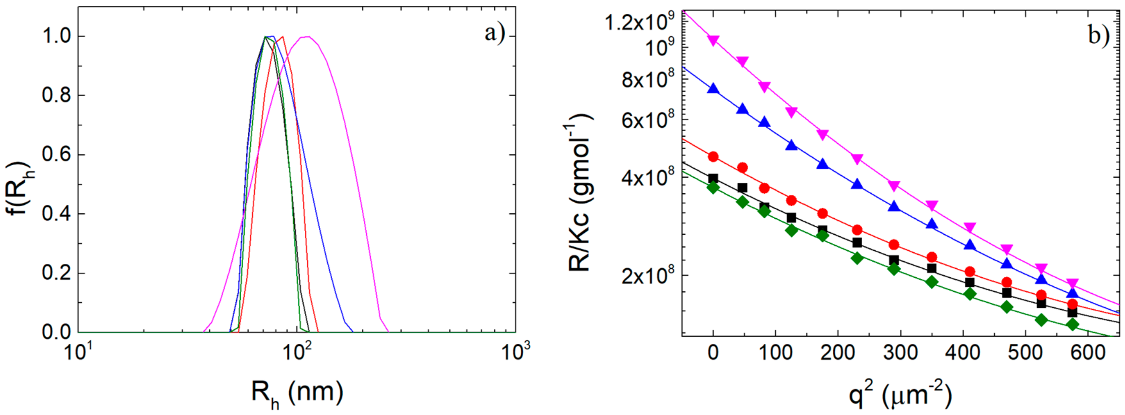

Figure S3. (**a**) CONTIN analysis at 90° and (**b**) Guinier plots of β-LG/CS complexes after TT at pH 4 for 0 (black), 5 (red), 10 (blue), 20 (magenta) and 50 (olive)% T80/β-LG mass ratios with T80 being added after TT.

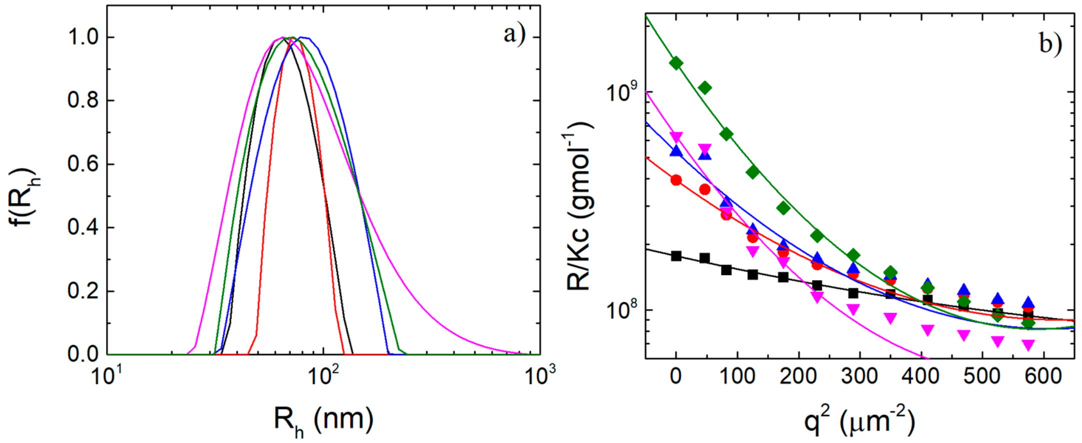

Figure S4. (**a**) CONTIN analysis at 90° and (**b**) Guinier plots of β-LG/CS complexes after TT at pH 4 for 0 (black), 5 (red), 10 (blue), 20 (magenta) and 50 (olive)% T80/β-LG mass ratios with T80 being added before TT.

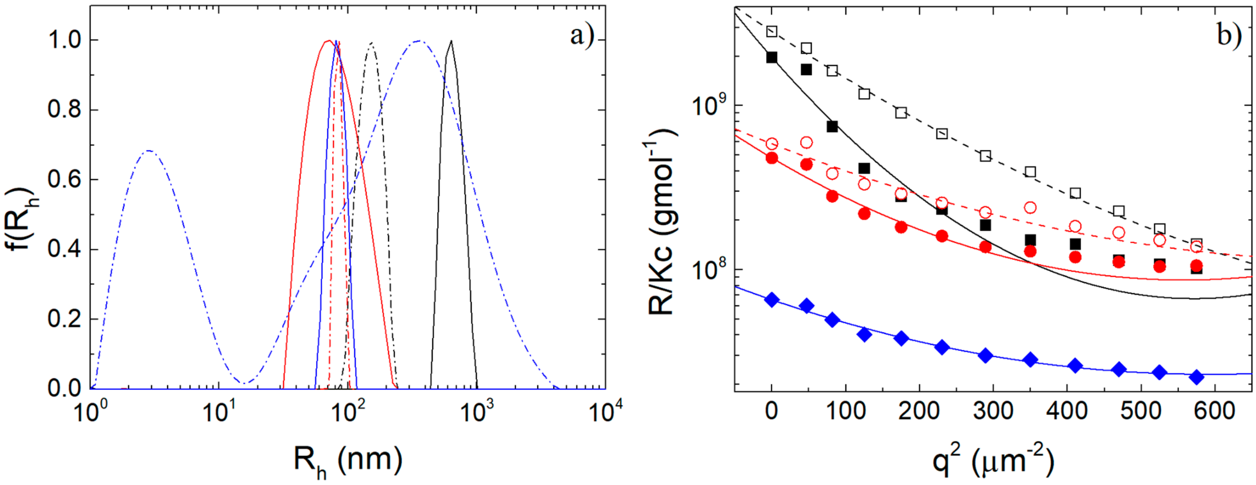

Figure S5. (**a**) CONTIN analysis at 90° and (**b**) Guinier plots of β-LG/CS complexes for 50% T80/β-LG mass ratio at pH 1.5 (black), 4 (red) and 7 (blue), before (dashed-dotted/dashed lines) and after (solid lines) TT (T80 added before TT).

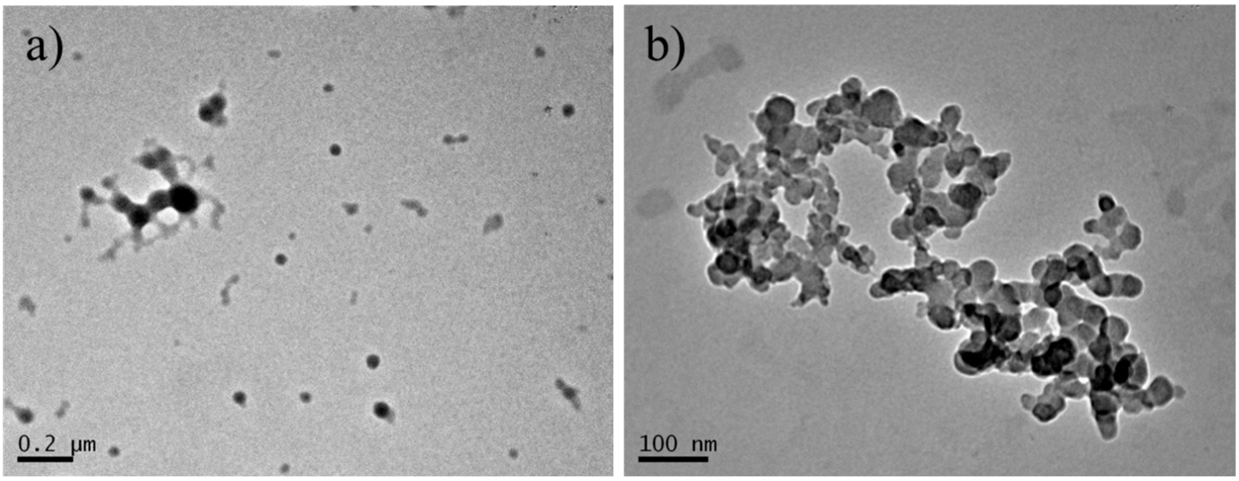

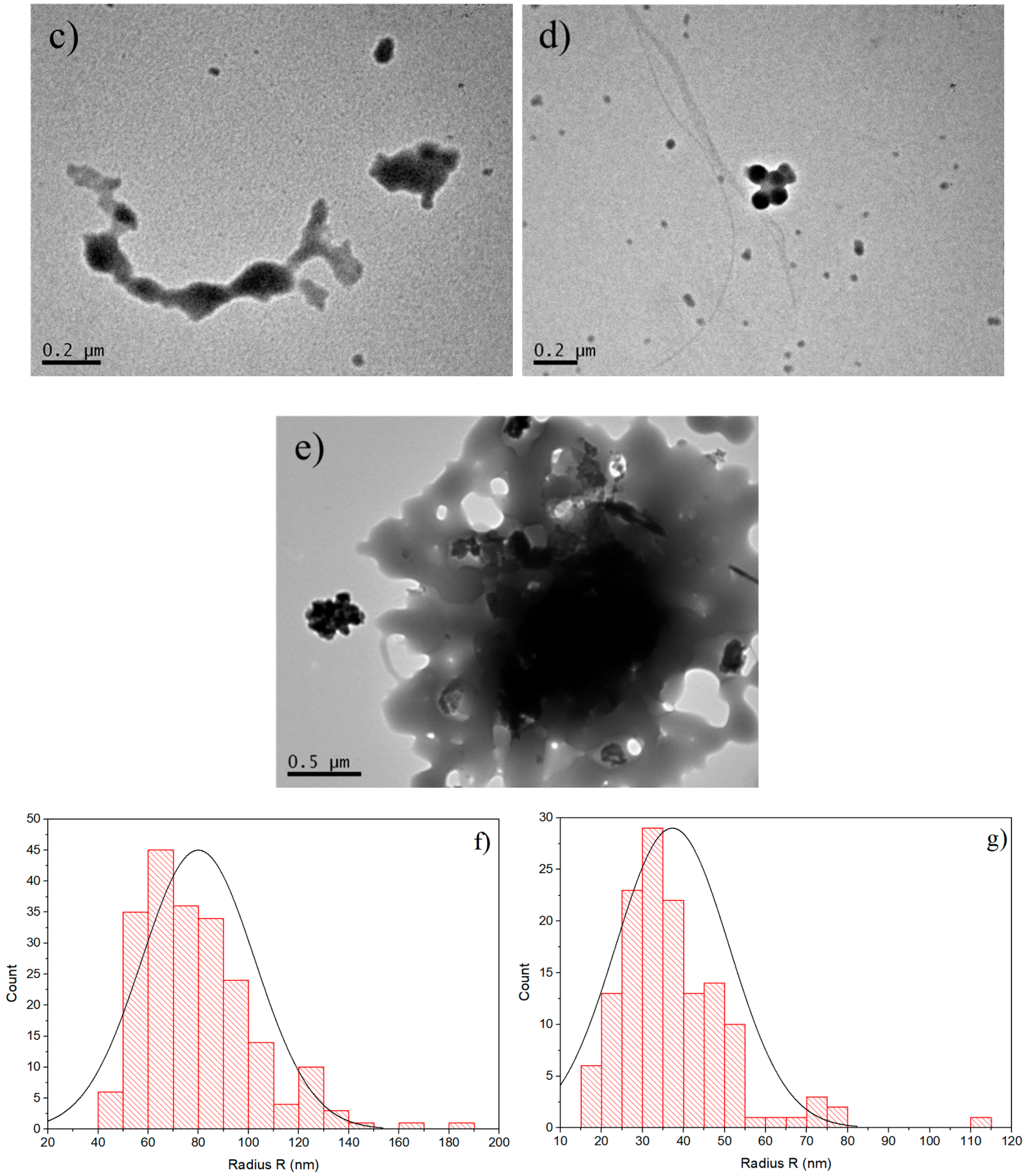

Figure S6. TEM images from clusters and aggregates of β-LG/CS NPs at 0.2 mg/mL β-LG (**a**) after TT at pH 4 and (**b**) at pH 7. TEM images from clusters and aggregates of β-LG/CS/T80 NPs at 0.2 mg/mL β-LG (**c**) before TT and (**d**) after TT at pH 4 and (**e**) after TT at pH 7. Size distributions of β-LG/CS NPs at pH 4 after TT (**f**) without and g) with T80 extracted from multiple TEM images (Gaussian fits are presented along the respected size distributions).

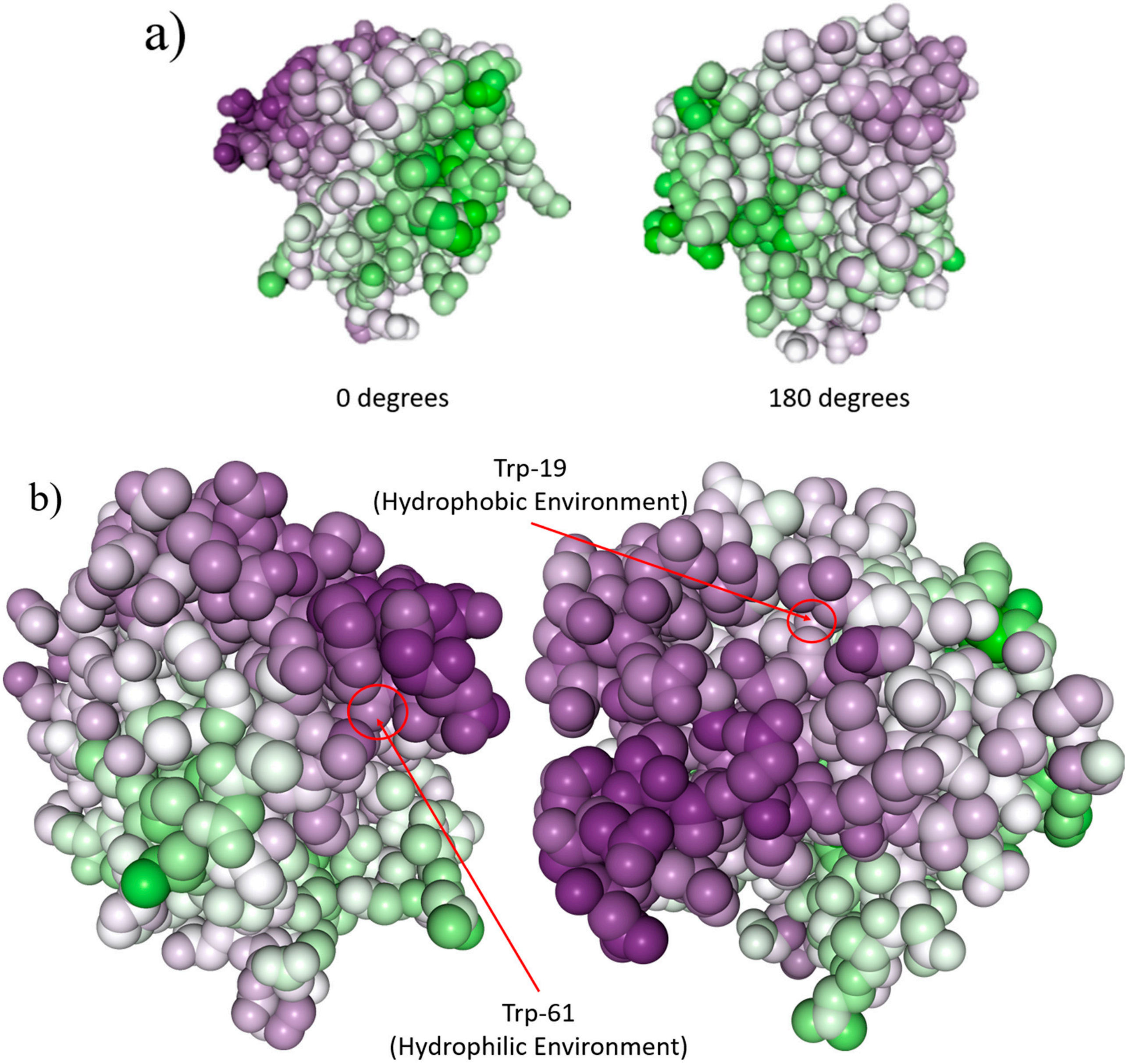

Figure S7. (**a**) Ratio of non-polar to polar sites of β-LG molecule at 0° and 180°. The ratio of non-polar/polar residues color scale is in the range between 0.6 (purple) and 2.3 (green). (**b**) Positions of Trp 19 and Trp 61 in plan view of the β-LG molecule respectively.

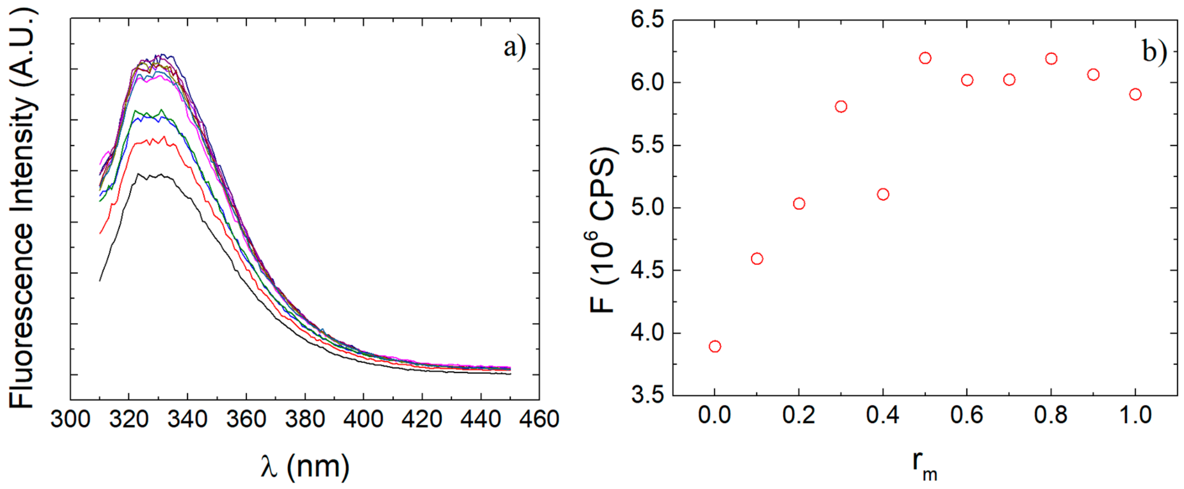

Figure S8. (**a**) Tryptophan fluorescence of β-LG with the addition in situ of CS in pure β-LG at pH 4 with r_m values of 0.0 (black), 0.1 (red), 0.2 (blue), 0.3 (magenta), 0.4 (green), 0.5 (navy blue), 0.6 (purple), 0.7 (wine), 0.8 (brown), 0.9 (olive) and 1.0 (cyan). (**b**) Maximum peak intensities in relation to r_m values for the case of the addition in situ of CS in pure β-LG at pH 4 (Maximum peaks at 328 nm were selected after smoothing the experimental fluorescence spectra).

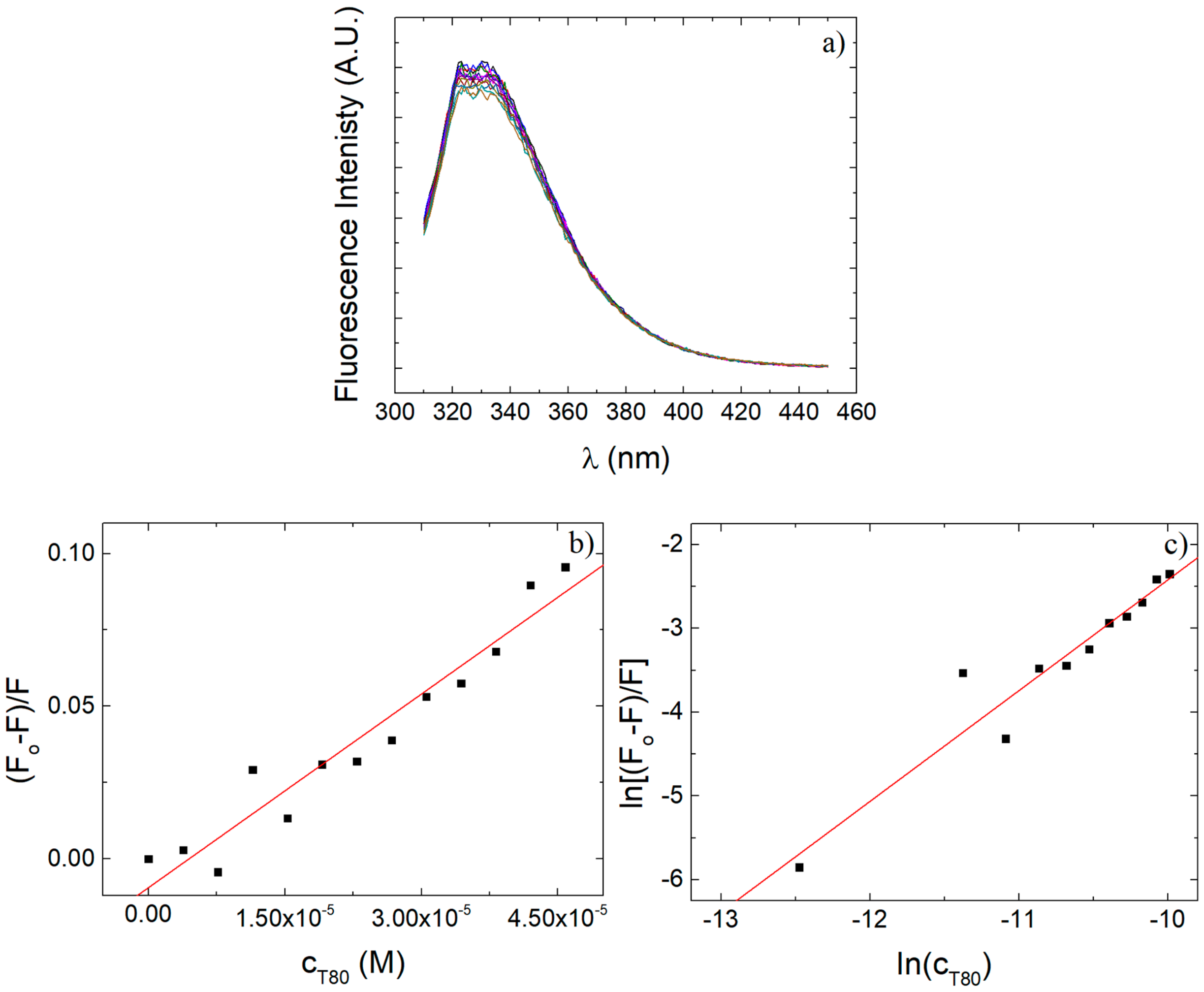

Figure S9. (**a**) Tryptophan fluorescence of β-LG with the addition in situ of T80 in pure β-LG at pH 4 with r_m values of 0 (black), 5 (red), 10 (blue), 15 (magenta), 20 (green), 25 (navy blue), 30 (violet), 35

(purple), 40 (wine), 45 (dark yellow), 50 (dark blue), 55 (cyan) and 60 (brown). Linear fits of the fluorescence intensity at maximum from titration of T80 at pH 4 for the evaluation of (**b**) the quenching constant K_{SV} and c) the binding constant K_A and the number of binding sites n per protein globule (Maximum peaks at 328 nm were selected after smoothing the experimental fluorescence spectra).

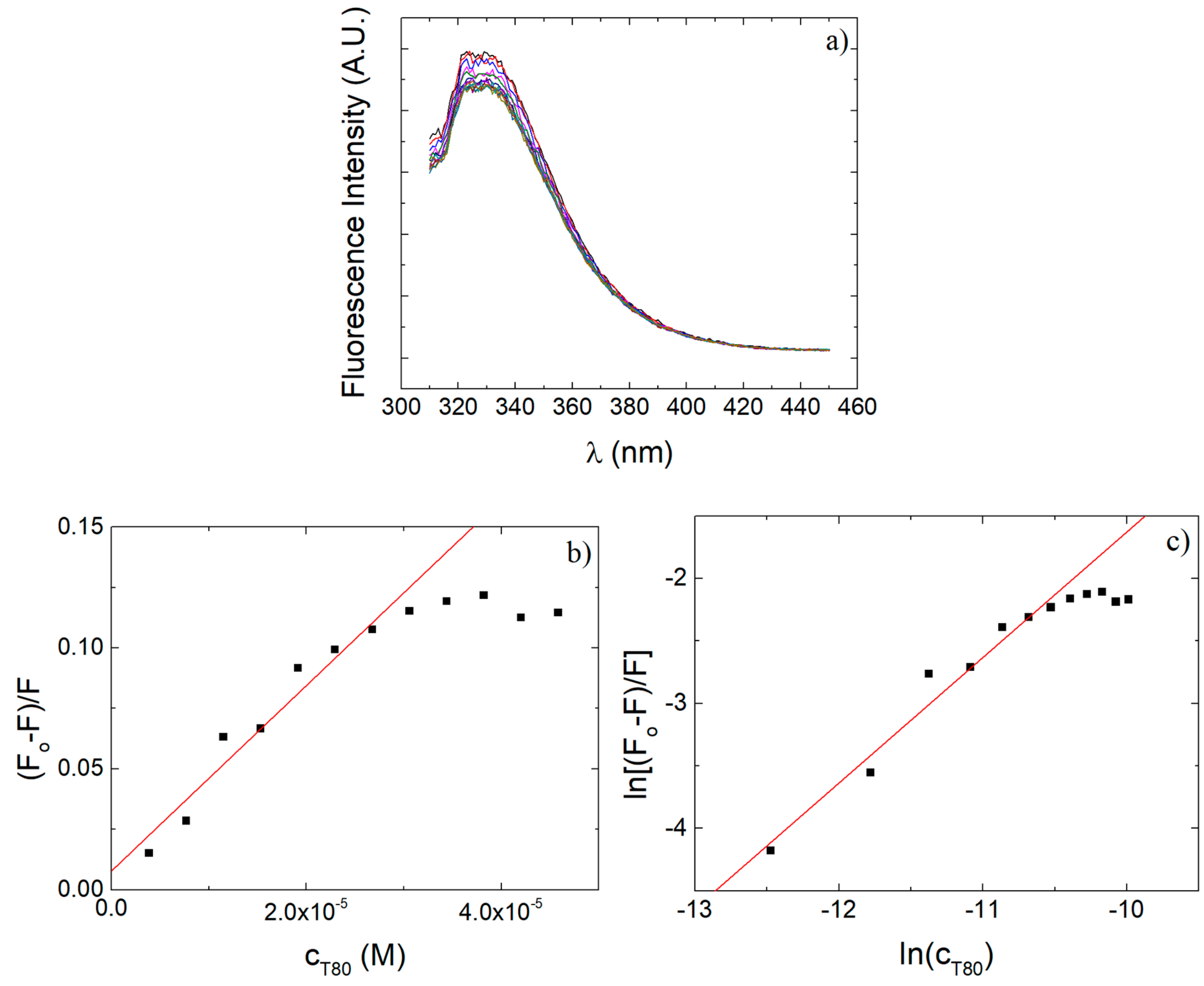

Figure S10. (**a**) Tryptophan fluorescence of β-LG with the addition in situ of T80 in β-LG/CS NPs at pH 4 with r_m values of 0 (black), 5 (red), 10 (blue), 15 (magenta), 20 (green), 25 (navy blue), 30 (violet), 35 (purple), 40 (wine), 45 (dark yellow), 50 (dark blue), 55 (cyan) and 60 (brown). Linear fits of the fluorescence intensity at maximum from titration of T80 at pH 4 for the evaluation of (**b**) the quenching constant K_{SV} and (**c**) the binding constant K_A and the number of binding sites n per protein globule (the last four points from the graphs are excluded from the linear fit due to intensity saturation) (Maximum peaks at 328 nm were selected after smoothing the experimental fluorescence spectra).

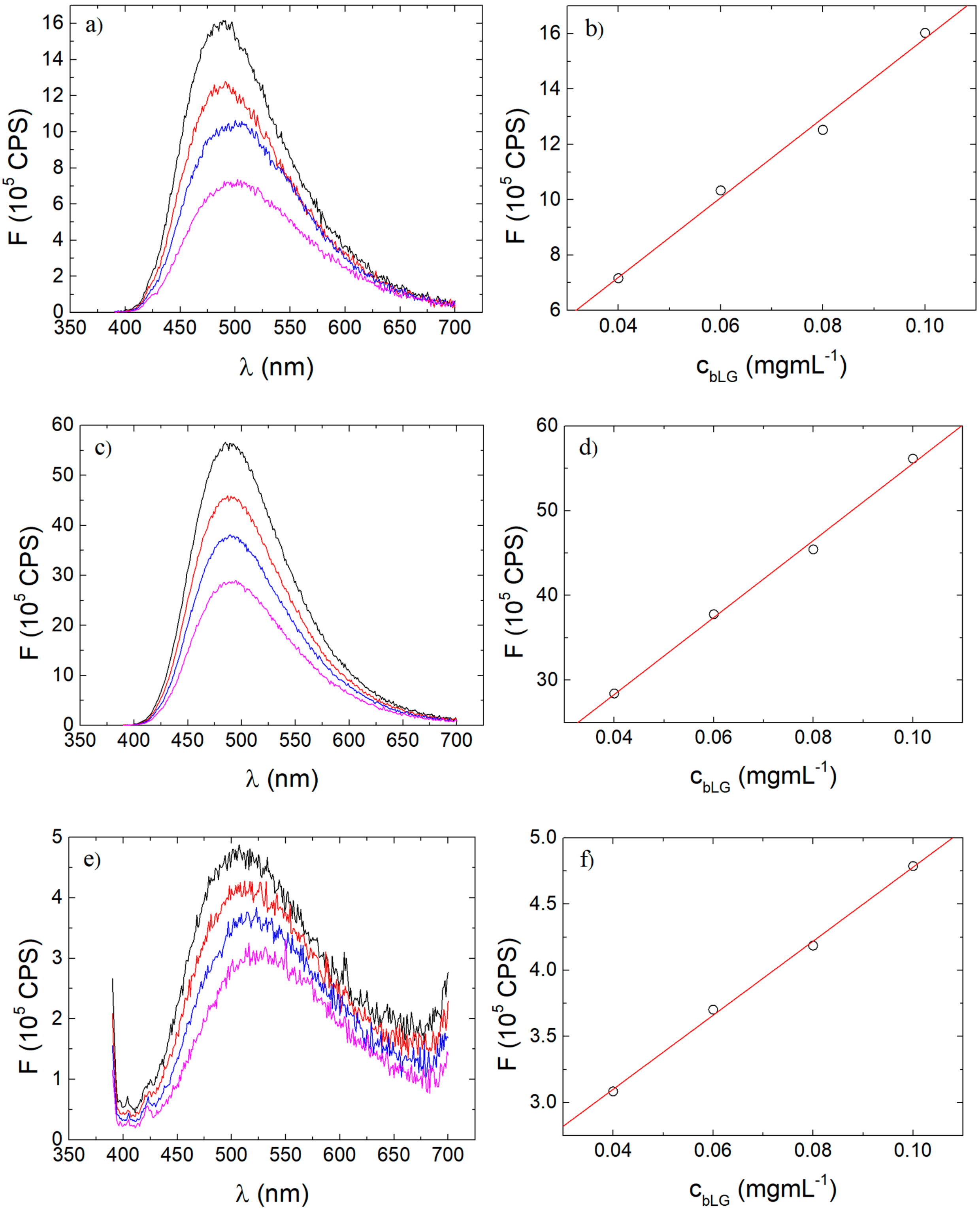
a)
b)
c)
d)
e)
f)
F (10^5 CPS)
λ (nm)
c_bLG (mgmL^-1)
0 2 4 6 8 10 12 14 16
6 8 10 12 14 16
0 10 20 30 40 50 60
30 40 50 60
0 1 2 3 4 5
3.0 3.5 4.0 4.5 5.0
350 400 450 500 550 600 650 700
0.04 0.06 0.08 0.10

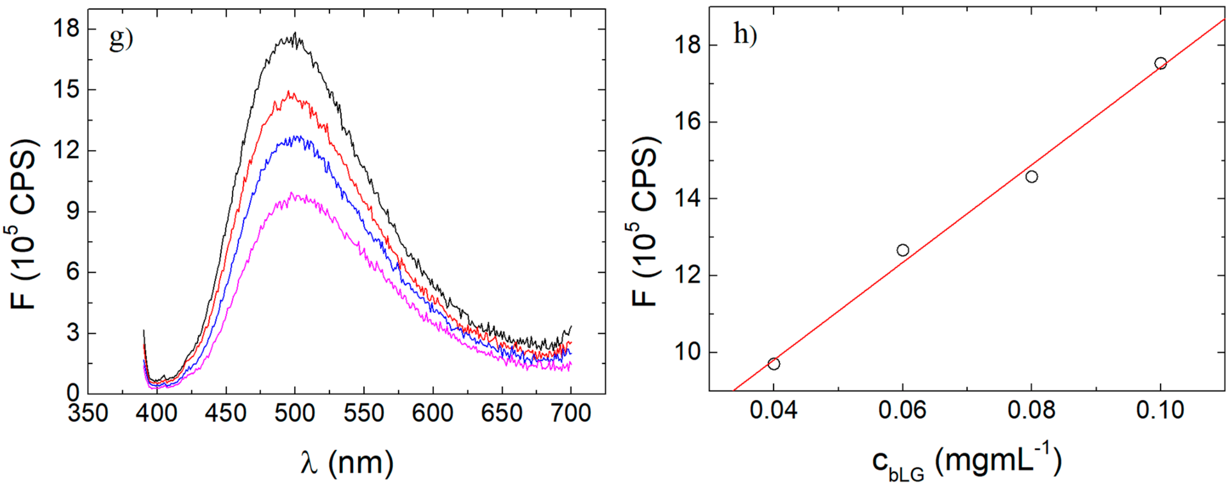

Figure S11. ANS fluorescence spectra of pure β-LG (**a**,**b**), pure β-LG/T80 (**c**,**d**), β-LG/CS (**e**,**f**), and β-LG/CS/T80 (**g**,**h**) NPs before TT. $C_{\beta\text{-LG}}$: 0.1 (black), 0.08 (red), 0.06 (blue) and 0.04 (magenta) $mgmL^{-1}$ (Maximum peaks at 496-505 nm were selected after smoothing the experimental fluorescence spectra).

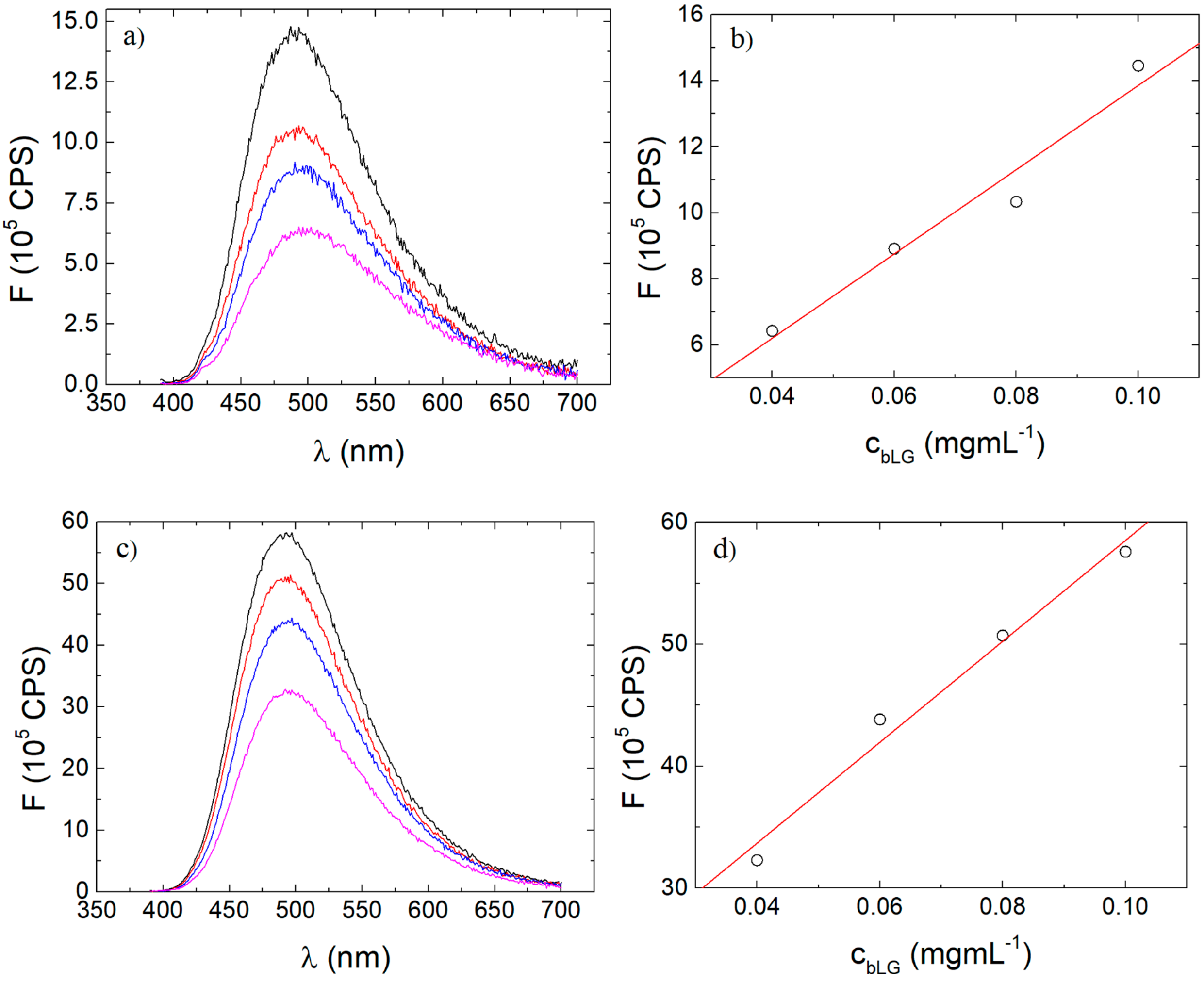

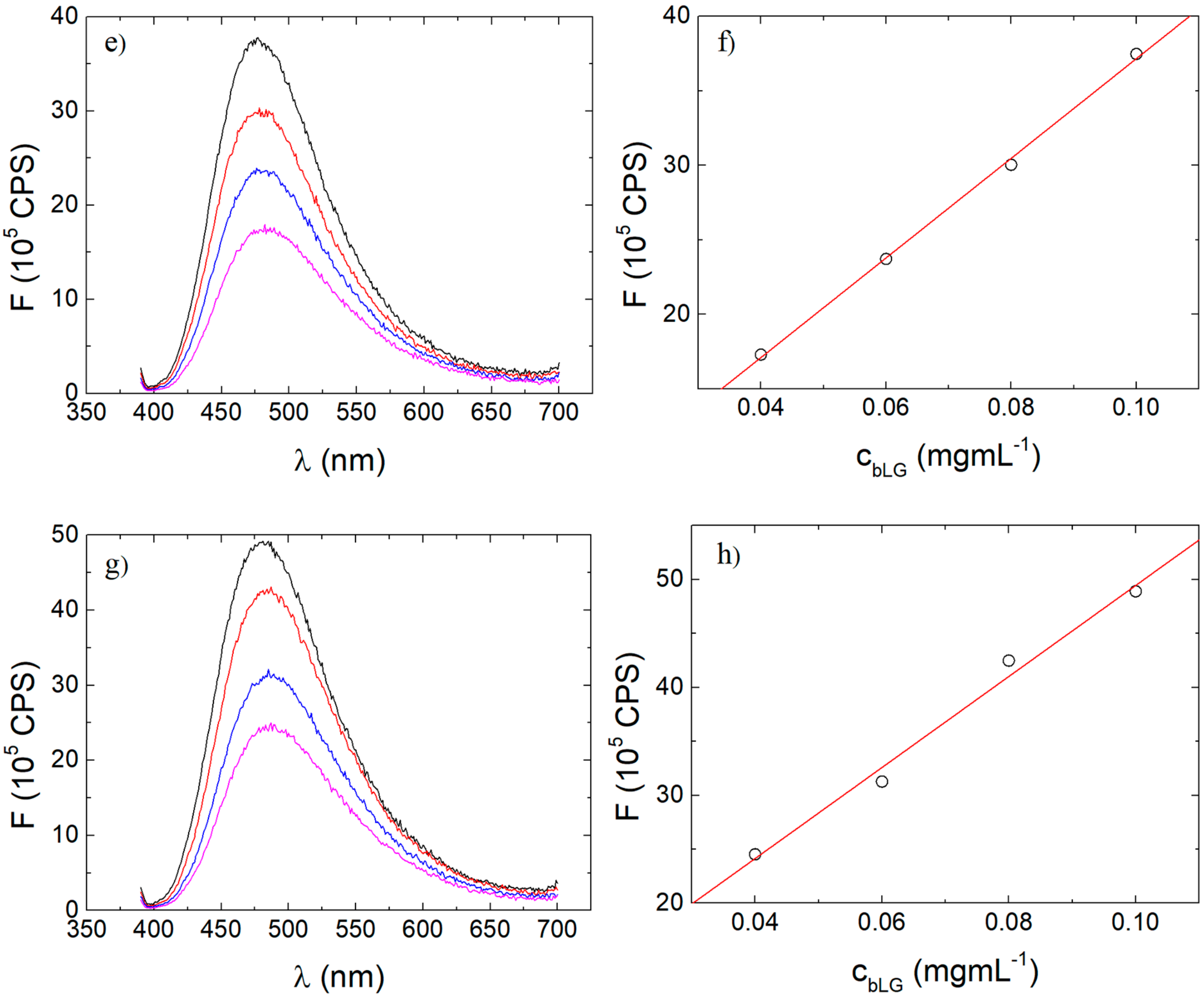

Figure S12. ANS fluorescence spectra of pure β-LG (**a**,**b**), pure β-LG/T80 (**c**,**d**), β-LG/CS (**e**,**f**), and β-LG/CS/T80 (**g**,**h**) NPs after TT. $C_{\beta\text{-LG}}$: 0.1 (black), 0.08 (red), 0.06 (blue) and 0.04 (magenta) mgmL^{-1} (Maximum peaks at 496-505 nm were selected after smoothing the experimental fluorescence spectra).

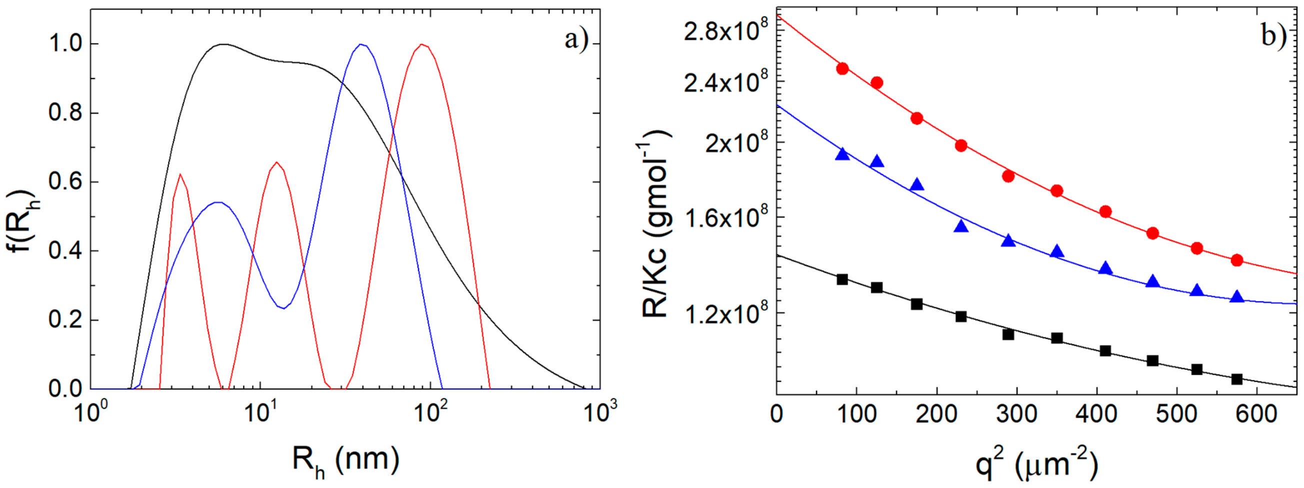

Figure S13. (**a**) CONTIN analysis at 90° and (**b**) Guinier plots for a reference sample with H_2O (black) an thermally treated β-LG/CS complexes at pH 7 with (blue) and without (red) T80 in the FBS:PBS solutions.

Table S3. Experimental results of a reference sample with H_2O and thermally treated β-LG/CS and β-LG/CS/T80 NPs in FBS:PBS solution at pH 7 (Uncertainties: δM_w=±15%, δR_g=±7%, δR_h=±5% and δϱ=±8%).

	SLS		DLS (Cumulant, 90°)		DLS (CONTIN, 90°)	
**Sample**	**M_w (gmol^{-1})**	**R_g (nm)**	**R_h (nm)**	**PDI**	**R_h (nm)**	**ϱ**
Reference	$1.43 \cdot 10^8$	51.5	11.1	0.486	17.9	2.88
β-LG/CS	$2.93 \cdot 10^8$	76.0	22.8	0.506	89.1 (59%)	0.85
					7.00 (40%)	-
β-LG/CS/T80	$2.24 \cdot 10^8$	72.7	15.9	0.515	18.8	3.87

Table S4. Molecular mass M_w, radius of gyration R_g, hydrodynamic radius R_h from CONTIN analysis in 90° and shape factor ϱ of β-LG/CS NPs at pH 4 with r_m 0.4 and 0.8 for day 0, 15 and 30 (Uncertainties: δM_w=±15%, δR_g=±7%, δR_h=±5% and δϱ=±8%).

Time	r_m	M_w (gmol^{-1})	R_g (nm)	R_h (nm)	ϱ
Day 0	0.4	$3.39 \cdot 10^8$	83.1	76.8	1.26
	0.8	$1.40 \cdot 10^8$	81.5	62.5	1.64
Day 15	0.4	$4.73 \cdot 10^8$	97.7	96.1	1.30
	0.8	$4.70 \cdot 10^8$	185	66.9	3.40
Day 30	0.4	$4.63 \cdot 10^8$	112	85.3	1.28
	0.8	$4.48 \cdot 10^8$	182	77.1	2.59

Table S5. Molecular mass M_w, radius of gyration R_g, hydrodynamic radius R_h from CONTIN analysis in 90° and shape factor ϱ of thermally treated β-LG/CS NPs with r_m 0.4 and at pH 1.5, 4 and 7 for day 0 and 20 (Uncertainties: δM_w=±15%, δR_g=±7%, δR_h=±5% and δϱ=±8%).

Time	pH	M_w (gmol^{-1})	R_g (nm)	R_h (nm)	ϱ
Day 0	1.5	$1.53 \cdot 10^9$	156	7000	0.02
	4.0	$3.39 \cdot 10^8$	83.1	76.8	1.26
	7.0	$1.32 \cdot 10^7$	193	101	1.91
Day 20	1.5	$6.69 \cdot 10^8$	116	6150	0.02
	4.0	$3.99 \cdot 10^8$	75.9	74.3	1.10
	7.0	$2.94 \cdot 10^7$	159	114	1.48

Table S6. Molecular mass M_w, radius of gyration R_g, hydrodynamic radius R_h from CONTIN analysis in 90° and shape factor ϱ of thermally treated (TT) and not (NoTT) β-LG/CS/T80 NPs (50% T80/β-LG mass ratio) with r_m 0.4 and at pH 4 for day 0 and 30 (Uncertainties: δM_w=±15%, δR_g=±7%, δR_h=±5% and δϱ=±8%).

Time	State	M_w (gmol^{-1})	R_g (nm)
Day 0	NoTT	$5.85 \cdot 10^8$	110
	TT	$3.72 \cdot 10^8$	82.9
Day 30	NoTT	$5.63 \cdot 10^8$	110
	TT	$1.36 \cdot 10^8$	92.8
